# Long read sequencing reveals novel isoforms and insights into splicing regulation during cell state changes

**DOI:** 10.1186/s12864-021-08261-2

**Published:** 2022-01-10

**Authors:** David J. Wright, Nicola A. L. Hall, Naomi Irish, Angela L. Man, Will Glynn, Arne Mould, Alejandro De Los Angeles, Emily Angiolini, David Swarbreck, Karim Gharbi, Elizabeth M. Tunbridge, Wilfried Haerty

**Affiliations:** 1 Earlham Institute, Norwich Research Park, Norfolk, NR4 7UZ UK; 2 Department of Psychiatry, Medical Sciences Division, University of Oxford, Oxfordshire, OX3 3JX UK; 3 Oxford Health, NHS Foundation Trust, Oxford, Oxfordshire OX3 7JX UK

## Abstract

**Background:**

Alternative splicing is a key mechanism underlying cellular differentiation and a driver of complexity in mammalian neuronal tissues. However, understanding of which isoforms are differentially used or expressed and how this affects cellular differentiation remains unclear. Long read sequencing allows full-length transcript recovery and quantification, enabling transcript-level analysis of alternative splicing processes and how these change with cell state. Here, we utilise Oxford Nanopore Technologies sequencing to produce a custom annotation of a well-studied human neuroblastoma cell line SH-SY5Y, and to characterise isoform expression and usage across differentiation.

**Results:**

We identify many previously unannotated features, including a novel transcript of the voltage-gated calcium channel subunit gene, *CACNA2D2*. We show differential expression and usage of transcripts during differentiation identifying candidates for future research into state change regulation.

**Conclusions:**

Our work highlights the potential of long read sequencing to uncover previously unknown transcript diversity and mechanisms influencing alternative splicing.

**Supplementary Information:**

The online version contains supplementary material available at 10.1186/s12864-021-08261-2.

## Introduction

The complex suite of processes that occur during transcription gives rise to a staggering diversity of protein structures, molecular interactions and cell fates. Alternative splicing (AS) allows different transcripts to be generated from a single gene. Differential transcript expression (the overall abundance of a given transcript) or transcript usage (the abundance of a given transcript relative to that of others produced from the same gene) are key mechanisms for regulating cell lineage commitment and function [1, 2]. In vertebrates, AS is particularly prominent in the brain, and regulates multiple aspects of neurodevelopment including neurogenesis, synaptogenesis, cellular migration and axon guidance [3–5] in a temporally precise manner [6–8]. These neurodevelopmental processes are defined by ordered switches in exon usage and expression across a spectrum of genes, controlled by a suite of highly specific RNA-binding proteins (RBPs) such as NOVA2 [9], PTBP1 and PTBP2 [10–12]. A number of more ubiquitous RBPs may also help regulate these neuronal AS events, though which ones and what specific roles they play remain poorly understood [13, 14].

Since AS can give rise to mRNAs that encode protein isoforms that exhibit distinct, or even opposing effects, it is essential to understand an individual gene’s products at transcript-level resolution [7, 15–17]. However, the diversity of full-length transcripts remains poorly understood, as exemplified by the recent study of the L-type voltage gated calcium channel (VGCC) gene, *CACNA1C* [18]. Furthermore, many unknowns remain as to the nature and regulation of changes in transcript expression during differentiation and development. For example, are there pronounced switches in primary transcript expression in a few key genes, or more nuanced expression differences across the transcriptome? Furthermore, although some of the molecular mechanisms that drive the observed ‘switches’ in transcriptional profiles occurring during lineage commitment have been identified, many of these processes remain to be determined.

As well as being of importance for understanding normal developmental processes, AS is also of clinical relevance, since aberrant transcriptional processes are implicated in many diseases [19]. Disease-associated mutations can directly affect AS by disrupting existing splice sites and/or forming novel or cryptic sites, as observed in the VGCC *CACNA1A* gene in Episodic Ataxia Type 2 [20]. Alternatively, AS can alter disease presentation, as is seen in the case of Timothy Syndrome where the localisation of the disease-causing mutation in one of two mutually exclusive exons of *CACNA1C* determines syndrome severity [21]. Global changes in differential isoform expression are also associated with psychiatric conditions [22].

Transcriptome profiling and annotation are essential first steps in investigating gene, isoform and exon expression or usage differences during cell differentiation. Until recently profiling was hampered by technological constraints, relying on short read sequencing technology [23, 24]. Whilst short read technologies provide cheap, accurate and high-coverage reads, with good differential expression analysis power [24], their ability to resolve and quantify full-length transcripts is inherently limited [25]. In this context, the advent of long read technologies has rapidly improved our ability to characterise the transcriptome [25, 26] revealing, for example, the complexity of the transcriptional landscape of the mammalian brain [27, 28].

Here, we use long read sequencing to identify and quantify isoforms during a cellular state change; specifically, during the differentiation of the well-validated SH-SY5Y neuroblastoma line into neuron-like cells. SH-SY5Y cells exhibit a stable genomic structure and have been widely used to investigate AS mechanisms and cellular differentiation from a neuroblast-like state [29–32], into a neuronal-like state [33, 34]. We generated a custom high-coverage long read transcriptome annotation (using Oxford Nanopore Technology [ONT] cDNA sequencing) and applied differential expression and usage analyses to uncover transcript variation during differentiation.

## Results

### ONT reads accurately detect differential isoform expression

**Fig. 1:**
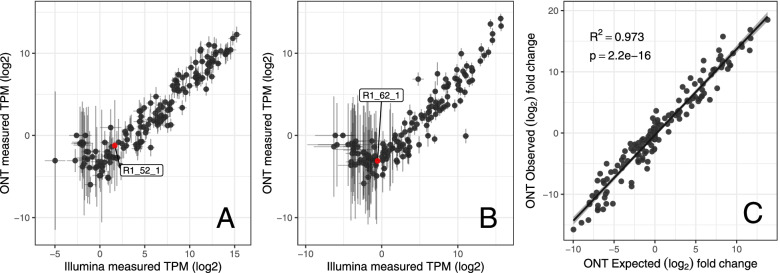
Comparison of Sequin spike-in sensitivity of detection between ONT (long read) and short read sequencing (Illumina paired-end, SRS) sequencing for Sequin synthetic spike-ins MixA (**A**) and MixB (**B**). Labelled sequin (red point) in each plot is at ONT limit of quantification (LOQ) in each mix; mix A 0.059 attomol/μl and mix B 0.27 attomol/μl. Plot **C** shows correlation of ONT differential isoform observed vs observed expression (log2 fold change + 1) of synthetic Sequin spike-ins of known concentration. Pearson correlation coefficient is displayed along with a linear regression trend line with standard error in pale grey

**Fig. 2:**
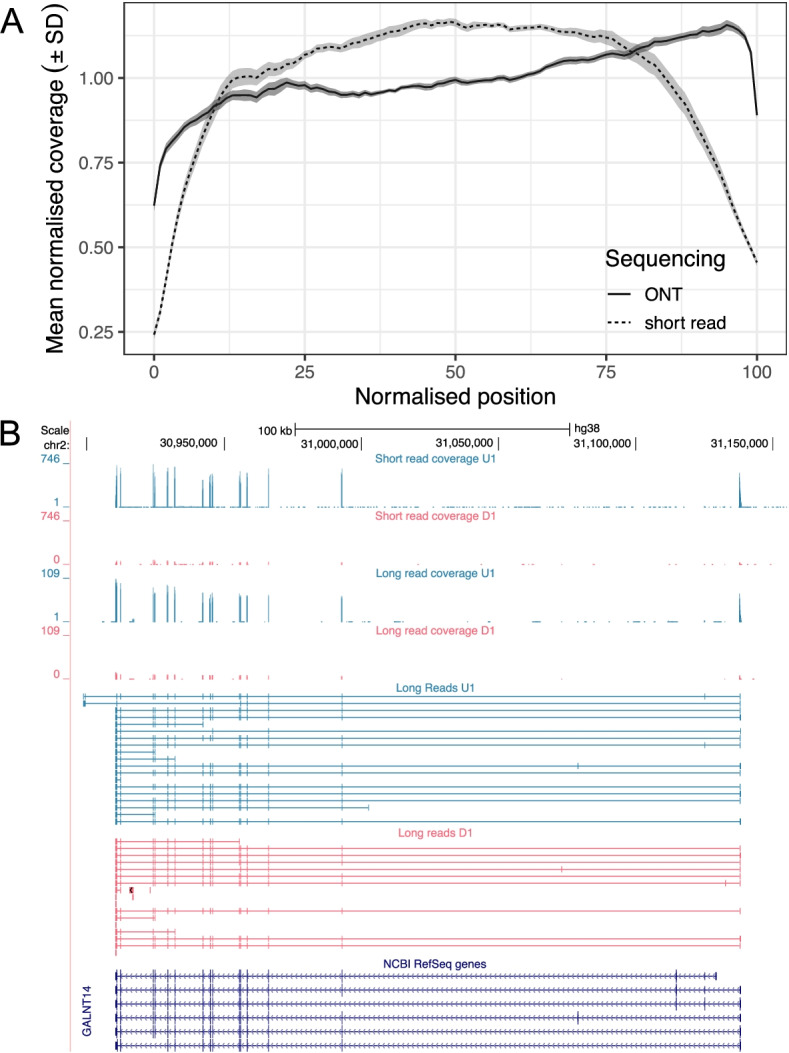
**A** Mean normalised coverage (± std. dev) across transcript normalised positions for ONT and Illumina libraries calculated with picard toolkit. **B** Custom UCSC Genome Browser visualization of the full coverage of short read RNA-Seq and a subset of long read RNA across a representative genome model for two samples (undifferentiated: blue, differentiated: red). Full UCSC tracks for visualisation of all sequencing reads are available as supplementary material

Using the Oxford Nanopore GridION platform, we generated on average 10,691,538 QC-passed reads per sample (± 1,751,518.6 SD). We also generated an average of 105,349,119 lllumina read pairs per sample (± 17,312,599.92 SD, Table S1). We investigated the ability of the ONT data to detect Sequin spike-ins [35] of known concentration in a set of two different concentration mixes. We found that the ONT limit of quantification (minimum transcript concentration) was 0.059 attomol/μl for mixA and 0.27 attomol/μl for mixB (Fig. 1A, B). By downsampling the short read data to the ONT average nucleotide coverage, we show there is similar power to detect transcripts by ONT and downsampled short read, although ONT scores lower than the complete short read data (Table S2). Next, we directly assessed the ability of the ONT reads to detect differential isoform expression. We calculated expected log_2_ fold-change (logFC) from the differences in concentrations between Sequins in mixA and mixB and compared this with observed logFC from our differential expression pipeline (see Methods). There was a strong correlation between expected and observed logFC of R^2^ = 0.973, *p*-value = 2.2e^− 16^ (Fig. 1C), demonstrating that the ONT data can be used to detect differential expression over a broad range of logFC values. Quantifying normalised coverage of both sequencing approaches on the whole dataset, we show minimal 5′ or 3′ bias in the short reads and limited 3′ bias in the ONT read sequencing (Fig. 2A). We also demonstrate the ability to detect full length transcripts with the ONT read and consistently detect differential expression (Fig. 2B).

### TALON custom long read annotation reveals novel features of the human transcriptome

**Fig. 3:**
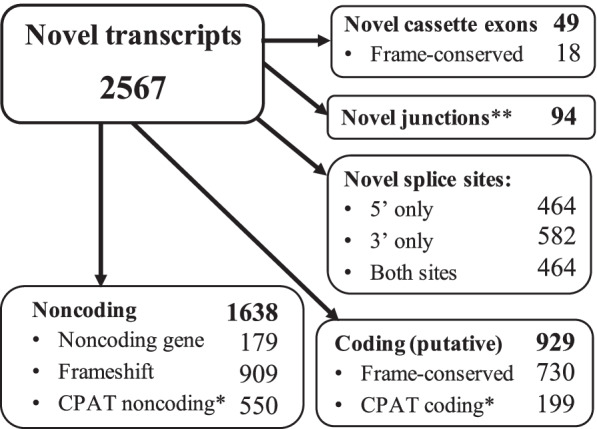
Breakdown of novel transcripts identified using ONT long reads and TALON custom transcriptome annotation. Cassette exons are previously unannotated positions. *CPAT assessment of CDS coding probability (CP ≥ 0.364). **Novel junctions are previously unannotated junctions identified between existing exonic parts. All novel assessments are relative to Gencode v29 human transcriptome annotation

The TALON custom annotation provided a total of 3274 novel transcripts prior to validation using short read sequencing. We found short read support for 2567 of the 3274 (78.41%) novel transcripts recovered from the ONT read data (Fig. 3) by stringent removal of transcripts that contained a novel exon lacking at least 15 reads depth across 75% of its length (see methods). The supported novel transcripts collectively include a total of 49 novel cassette exons (18 frame-conserving) along with 928 and 1046 novel 5′ and 3′ splice sites respectively, with 464 instances of exons exhibiting both a novel 5′ and 3′ splice site. Using our short read sequencing data we validated 1019 out of 1520 novel junctions. Additionally, we identified 92 novel junctions between previously annotated splice sites. In total 929 (36.19%) of the validated novel transcripts were putatively coding; either frame-conserving, or assumed to be coding via CPAT [36] assessment, whilst 1638 (63.81%) were assumed noncoding due to either induction of a frameshift, a noncoding parent gene or via noncoding classification from CPAT (Fig. 3 for full breakdown). Finally, of the 2567 novel transcripts, 983 were found to possess novel transcription start sites compared with known transcripts in the reference GTF. Intersecting these against Fantom5 CAGEseq data [37] revealed a total of 824 peaks overlapping the novel start sites (± 500 bp) of 333 novel transcripts (see Table S6).

Using this custom annotation, we were able to quantify genome-wide alternative splicing events, and revealed significant differences between the cell states in categories such as alternative transcription start and termination sites, and intron retention (see Table S7).

### ONT differential gene expression supports neuron-like characteristics of differentiated SH-SY5Y cells

**Fig. 4:**
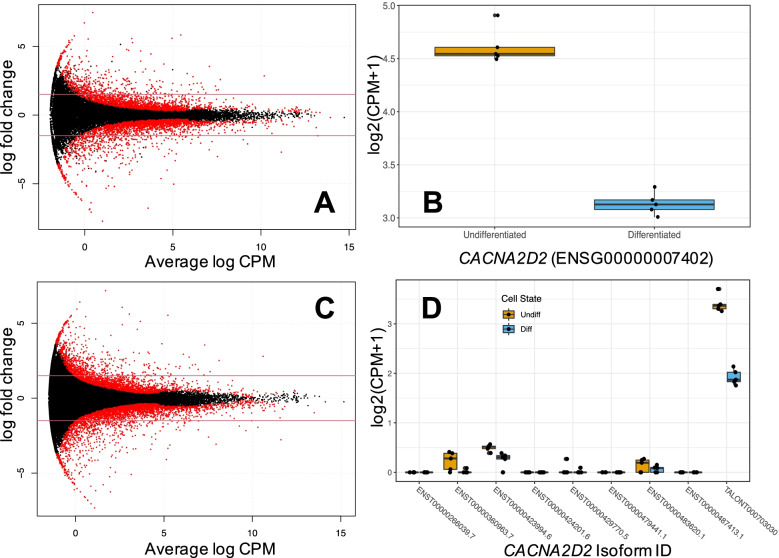
Panel of **A** gene-level differential expression (DE) smear plot, solid red lines highlighting ±1.5 logFC threshold, **B** gene-level DE of *CACNA2D2* during differentiation, **C** isoform-level DE smear plot with ±1.5 logFC threshold, **D** *CACNA2D2* isoform expression, showing novel TALON isoform with highest read count. Red points on smear plots indicate significant differential expression (FDR < 0.05). Boxplots display median and IQR. Short and long read mapping example provided in Fig. S8

Differential gene expression analysis revealed 4239 genes differentially expressed (false discovery rate [FDR] < 0.05) between differentiation states, with 2041 and 2198 genes overexpressed in undifferentiated and differentiated cells, respectively (Table 1, Fig. 4A). The gene ontology analyses revealed the upregulated genes in the differentiated cells showed greatest overlap with those upregulated in brain compared with other tissue types (p_adj_ = 3.4 × 10^− 41^), whilst for those more highly expressed in undifferentiated cells, there was overlap with those downregulated in brain (p_adj_ = 2.8 × 10^− 45^, second only to pancreas: p_adj_ = 1.8 × 10^− 45^). Gene ontology biological pathway terms showing differential expression across differentiation included neurogenesis, neuron development, cell differentiation and regulation of nervous system development (see supplementary data for full FUMA results).

**Table 1:** Showing number of genes and transcripts differentially expressed between undifferentiated and differentiated cells, after multiple testing correction using ONT long read counts (FDR < 0.05). Bracketed numbers refer to the portion of total that are TALON-identified novel transcripts. U = undifferentiated and D = differentiated cells, with arrows displaying expression directionality

Metric	Count	
	Gene level	Transcript level (Talon)
Total features assessed	32,977	99,067 (1855)
Differentially Expressed	4239	5456 (197)
↑U ↓D (all > 0 log_2_FC)	2041	2390 (67)
↑D ↓U (all < 0 log_2_FC)	2198	3066 (130)

### Isoform-level differential expression reveals novel diversity

At the isoform-level, we detected differential expression of 5456 transcripts (FDR q-value < 0.05) in 4416 genes (Table 1, Fig. 4C) across cell state. It is important to note that, of the 4416 genes including differentially expressed isoforms, 1276 were not differentially expressed at the gene level. Gene ontology analysis of the genes that show differential transcript expression (DTE) in the absence of differential gene expression (DGE) identified intracellular trafficking and macromolecule localisation, and post-transcription and -translation processing mechanisms.

### Long read transcriptome annotation reveals a novel CACNA2D2 isoform and exon

As a specific example of the power of long read sequencing for uncovering isoform diversity, we identified a novel transcript (TALONT000703030) of the VGCC α_2_δ_2_ subunit gene, *CACNA2D2*, which is truncated (18,731 bp), compared to most previously annotated isoforms (87,831 bp - 141,442 bp, but note ENST00000483620 at 2927 bp, Fig. S2). This transcript is designated novel as it includes a novel first exon (chr3:50381499–50,381,529), with a potential in-frame start site (chr3:50381507–50,381,509) leading to an open reading frame. Orthogonal short read validation showed that this is not an artefact of ONT sequencing (Figs. S3, S7). Evidence for the existence of the exon can also be found in human cortex GTEx v.8 [38] RNA-seq data (*N* = 27 samples assessed from *N* = 21 individuals, Fig. S4) suggesting it is not unique to SH-SY5Y cells. Further validation is provided by successful RT-PCR of the novel exon and junction in all 10 samples (Fig. S7). Surprisingly, TALONT000703030 was the most highly expressed *CACNA2D2* isoform in the undifferentiated state and was downregulated in differentiated cells (Fig. 4D & isoform differential expression results), consistent with an overall downregulation of *CACNA2D2* at the gene level following differentiation (Fig. 4B & gene differential expression results).

### Differential Transcript Usage (DTU) analysis reveals splicing regulators relevant to SH-SY5Y differentiation

Whilst isoform-level expression aids the identification of important proteins and functional changes during differentiation, understanding differential transcript usage (i.e. the abundance of a given transcript relative to that of others produced from the same gene) may provide further insights. Our DTU analysis identified 104 isoform switches (each affecting a single gene) considered to have putative functional consequences (see methods) across SH-SY5Y differentiation (Table S4). The implicated genes include the histone deacetylase gene, *HDAC4*, and *RACGAP1*, which encodes Rac GTPase Activating Protein 1, a protein critical for axon morphogenesis [39]. We also identified RNA binding motif protein 5 (*RBM5*), a ubiquitous splicing regulator [40], among the genes showing differential transcript usage. Upon differentiation, we observed a switch from a truncated, non-coding *RBM5* isoform ENST00000474470 to the full-length coding isoform ENST00000347869 during differentiation for this splicing factor.

## Discussion

Utilising a long-read sequencing approach, we generated a custom high-coverage transcriptome annotation (using Oxford Nanopore Technology [ONT] cDNA sequencing), validated with orthogonal short read sequencing (Illumina paired-end short read) data. We identified novel transcriptomic features and performed differential expression and usage analyses to identify transcripts that show variation during differentiation of SH-SY5Y cells. Whilst the utility of long read sequencing for recovering full length transcripts is widely accepted, there remains uncertainty as to the sensitivity of this technology for differential expression analysis. It is therefore important to assess the performance of long read vs short read sequencing in both transcript quantification and its application to differential expression studies [28]. Collectively, our results indicate that our ONT data are sufficiently sensitive and powered to detect all but the lowest concentration transcripts and, further, that the ONT reads are suitable for differential isoform expression and usage analysis (Figs. 1 and 2). These results add to the growing literature highlighting the importance of assessing suitability of long read sequencing for differential expression. Further, we support and encourage the use of synthetic spike-in controls to accurately determine experimental sensitivity.

Utilising the long read data, we were able to uncover thousands of novel transcripts, supported by our orthogonal short-read sequencing. Our stringent filtering criteria and validation likely results in an underestimate of the true quantity of novel features present. A recent study utilising long read sequencing to identify transcriptome variation across different human tissues proposed nearly 100,000 novel transcripts [41]. This work employed a different analytical approach to different data and is not directly comparable, but it clearly demonstrates that our findings are well within a realistic and conservative scale. Our long read sequencing approach still identifies > 2500 novel transcripts, nearly a thousand of which are putatively coding. Further, we uncover evidence of Fantom5 CAGE peak support for 333 of 983 novel transcripts that possess putative novel start sites. There are several potential explanations for not finding peaks overlapping the other 650 such as the transcript expression level, sequencing depth and tissue specificity. Despite these limitations, we still find further external validation of a significant proportion of the novel transcripts detected. We additionally find some significant differences in AS events between the cell states such intron retention or alternative transcription termination sites (Table S7), but in all cases the effect sizes are small and inference of general patterns is not appropriate. Moreover, it suggests many, diverse changes as opposed to any particular AS mechanism(s) playing a dominant role. This work implicates a need for future functional assessment on an individual gene basis. Collectively, our data highlight the extent of previously undescribed transcriptome diversity, even within a highly specialised (and well-studied) cell model. Our work concurs with the growing body of other studies using long reads for transcriptome assessment [28, 42, 43]; that relying on short reads substantially underestimates transcriptome diversity.

The differential expression analyses provided insight into the differentiation process identifying thousands of both genes and isoforms involved. Previous studies have investigated differential gene expression during differentiation of SH-SY5Y cells, but often in relation to transfected [34] or treated lines [44] and largely limited to gene-level inferences, finding hundreds of genes differentially expressed with microarrays or short-read cDNA. With our long read coverage and good sample size we are able to achieve a high resolution view of the differentiation process (see supplementary materials for full differential expression results). A key finding of our analyses is that many isoform-level differential expression events are undetectable at gene-level. Analysing expression at the transcript level therefore provides a more accurate overview of transcriptional dynamics. Our findings highlight the importance of post-transcriptional mechanisms in cellular differentiation and development and emphasise the importance of understanding changes at the transcript level, as key processes may be obscured at the gene level.

The discovery of a novel *CACNA2D2* exon and transcript is of particular interest given the vital role VGCCs play in neuronal function. The CACNA2D2 encoding subunit is vital to normal channel trafficking and has complex regulatory effects on VGCC currents [45, 46]. Mutations in *CACNA2D2* have long been studied for links with epileptic and cerebellar ataxic phenotypes [47, 48], and previously described truncated proteins of CACNA2D2 resulting from mutations exhibit abnormal function [49]. We present multiple sources of evidence for the novel exon; from our sequencing approaches, RT-PCR validation and from publicly available data, which suggest the features are neither a sequencing artefact nor SH-SY5Y specific. It remains to be determined whether the novel isoform presented here is functional and, if so, how the function differs from annotated isoforms. A CPAT [36] analysis reveals it to be potentially coding and initial Phyre2 [50] predictions suggest it would result in a cleaved protein structure (Fig. S5). Alternative splicing of other VGCC subunits is a key means of generating functionally distinct channels [51]. However, the novel isoform lacks the signal peptide (SignalP 5.0, [52]) required for membrane insertion, and therefore is unlikely to encode a functional VGCC α2δ subunit [53]. Given that this transcript is downregulated as cells differentiate into neuron-like cells, it is possible that the switch from a non-functional to a functional ɑ2δ subunit represents a means of regulating VGCC signalling, potentially preventing its inadvertent activation in undifferentiated cells.

## Conclusions

Together, our findings demonstrate the power of long read sequencing for the characterisation and quantification of genes at the isoform level. We demonstrate that current annotations remain far from complete, even in the well-studied SH-SY5Y cell line. We show the utility of this model system and our technical approach for studying fundamental molecular processes underlying changes in cell state. Finally, our findings provide evidence of novel features in a key channel protein CACNA2D2 and identify candidates with differential usage profiles that may be useful avenues for future functional studies, to further understand the molecular mechanisms coordinating these changes in cell state.

## Materials and methods

### Sampling and sequencing

#### Cell culture and neuronal differentiation

A total of 10 technical replicates of human neuroblastoma SH-SY5Y cells were cultured in neurobasal media (Gibco 21,103–049) supplemented with B-27 Plus (ThermoFisher). Retinoic acid (SigmaAldrich) was added to five replicates to a final concentration of 10 mM, to induce cell differentiation to a neuron-like state; whilst five replicates were cultured to confluence in standard media. Cells were washed with phosphate buffered saline and harvested in QIAzol (Qiagen) to preserve RNA, before being stored at − 80 °C until RNA extraction.

#### RNA extraction and spike-in control

Total RNA was purified from the 10 replicate cell cultures using a Direct-zol RNA Miniprep Plus kit (Zymo Research), according to the manufacturer’s instructions. Internal controls were employed to assess long read sequencing sensitivity to transcript detection and differential expression. Sequin synthetic spike-ins [35] are designed in a range of sizes and utilised in two separate mixes (MixA v.2 & MixB v.1) of known, contrasting concentrations (see data repository for details). These were spiked into the 10 replicates in an alternating fashion, so that MixA and MixB were represented in both cell states to enable internal control. The Sequins were spiked in at 1% of the total RNA input amount (https://www.sequinstandards.com/). These were added to the native RNA prior to reverse transcription, as per manufacturer’s instructions.

#### cDNA long read sequencing (ONT)

Two hundred nanogram total RNA per sample was processed using the cDNA-PCR Sequencing Kit (Oxford Nanopore Technologies, SQK-PCS109). The first-strand reaction was prepared according to the ONT cDNA-PCR Sequencing protocol provided by the manufacturers (SQK-PCS109, https://community.nanoporetech.com/protocols/cdna-pcr-sequencing_sqk-pcs109/v/PCS_9085_v109_revJ_14Aug2019?devices=gridion). Post first-strand, the reaction was snap cooled and then incubated to 42 °C while 8 μl of second-strand switching primer (SSP) master mix was added (prepared according to the protocol). This was mixed and incubated at 42 °C for 2 min. One microlitre of Maxima H reverse transcriptase was then added to the second-strand reaction, maintained at 42 °C. The whole second-strand reaction was then mixed and incubated at 42 °C for 90 min. The reaction was then inactivated at 85 °C for 5 min and held at 4 °C until ready to proceed to PCR. After reverse transcription, the reaction was split into four aliquots in preparation for four replicate PCR tubes to generate sufficient products while minimising the risk of over-amplification. The reagents for PCR were prepared according to the cDNA-PCR Sequencing (SQK-PCS109) protocol and incubated under the following conditions; initial denaturation at 95 °C for 30 s followed by 15 cycles of: denaturation at 95 °C for 15 s, annealing at 62 °C for 15 s, extension at 65 °C for 5 mins; with a final extension of 65 °C for 6 mins and hold at 4 °C. Post-PCR each PCR aliquot was exonuclease I (NEB) treated. Aliquots were then pooled per sample and bead concentrated (Beckman Coulter, AMPure XP, A63880) prior to sequencing. The cDNA was quantified using High Sensitivity Qubit assays (ThermoFisher, Q32854) and sized using the 2100 Bioanalyzer instrument (Agilent Technologies, cat. no. G2939BA) High Sensitivity DNA assay (Agilent, 5067–4626). Two hundred fifty nanogram cDNA per sample was prepared for rapid adapter ligation (approximately 190 fmol per sample).

MinION flowcells underwent QC prior to library construction. The PCR cDNA libraries were prepared for sequencing and the respective MinION flowcells were primed following the cDNA-PCR Sequencing (SQK-PCS109) protocol using the PromethION flow cell priming kit (Oxford Nanopore Technologies, EXP-FLP001.PRO.6). Five PCR-cDNA libraries were sequenced in parallel per run of the GridION instrument with GridION software v.18.12.4 (Oxford Nanopore Technologies), using one Flowcell per library. Basecalling for all PCR-cDNA libraries was completed in real-time using MinKNOW on the GridION and maximum data acquisition time of 48 h was conducted for all 10 flowcells. ONT fast5 files were first converted to fastq using guppy v.3.2.2 (https://community.nanoporetech.com) before QC using MultiQC [54].

#### cDNA short read sequencing

Illumina library preparation and sequencing were carried out by the Genomics Pipelines team at Earlham Institute. One microgram of total RNA per sample was processed using the NEBNext Ultra II Directional RNA library prep kit from NEB (E7760L) utilising the NEBNext Poly(A) mRNA Magnetic Isolation Module (E7490L) with NEBNext Multiplex Oligos for Illumina® (96 Unique Dual Index Primer Pairs, E6440S) at a concentration of 10 μM. The RNA was purified to extract mRNA with a Poly(A) mRNA Magnetic Isolation Module. Isolated mRNA was then fragmented and cDNA was synthesised for the first strand. The second strand synthesis process removes the RNA template and synthesises a replacement strand to generate ds cDNA. Directionality is retained by adding dUTP during the second strand synthesis step and subsequent cleavage of the uridine-containing strand using USER Enzyme (a combination of UDG and Endo VIII). NEBNext Adaptors were ligated to end-repaired, dA-tailed DNA. The ligated products were subjected to a bead-based purification using Beckman Coulter AMPure XP beads (A63880). Adaptor ligated DNA was then enriched by receiving 10 cycles of PCR; 30 s at 98 °C, 10 cycles of: 10 s at 98 °C, 75 s at 65 °C, 5 mins at 65 °C, final hold at 4 °C. Barcodes were incorporated during the PCR step. The resulting libraries underwent QC using PerkinElmer GX and a High Sensitivity DNA chip (5067–4626), the concentration was determined with a High Sensitivity Qubit assay (Q32854) or plate reader. The final libraries were pooled, a qPCR was performed using a KAPA Illumina ABI library quantification kit (Roche Diagnostics, 7,960,204,001) on a StepOne q-PCR machine (ThermoFisher), and then these were prepared for sequencing.

The library pool was diluted to 0.65 nM with 10 mM Tris (pH 8.0) in a volume of 18 μl before spiking in 1% Illumina PhiX Control v.3. This was denatured by adding 4 μl 0.2 N NaOH and incubating at room temperature for 8 mins, after which it was neutralised by adding 5 μl 400 mM Tris (pH 8.0). A master mix of EPX1, EPX2, and EPX3 from Illumina’s Xp 2-lane kit was made and 63 μl added to the denatured pool leaving 90 μl at a concentration of 130 pM. This was loaded onto a NovaSeq S1 flow cell using the NovaSeq Xp Flow Cell Dock. The flow cell was then loaded onto the NovaSeq 6000 along with a NovaSeq 6000 S1 cluster cartridge, buffer cartridge, and 200 cycle SBS cartridge. The NovaSeq had NVCS v.1.6.0 and RTA v.3.4.4 and was set up to sequence 100 bp PE reads. The data were demultiplexed and converted to fastq using bcl2fastq2 v.2.20 (Illumina). Illumina data underwent QC with MultiQC v.1.5 [54] and adaptors were removed with trim galore v.0.5.0 [55] with default parameters. The 5′ and 3′ bias of both sequencing approaches were checked and compared by mapping normalised coverage and transcript normalised position for the whole dataset (10 samples per sequencing technology) using picard toolkit [56].

#### Custom transcriptome annotation and validation

The cDNA ONT reads were aligned to the human genome (hg38, modified to include an artificial chromosome containing the sequins spike-ins as contiguous transcripts) using minimap2 v.2.17 [57] in sam MD-tag aware mode. Only primary alignments were retained. We then employed TALON v.5.0 [58], a technology-agnostic pipeline that leverages long reads to build a custom transcriptome annotation. By exploiting the ability of long reads to detect full-length transcripts, TALON identifies novel features through comparison to an existing reference annotation. First, the sam files were passed to TranscriptClean v.2.0.2 [59] for correction of read microindels (< 5 bp) and mismatches, though any non-canonical splice junctions were retained for downstream analyses, as novel features can often be found with non-canonical junctions [60]. The reads were checked for internal priming artifacts, a known issue with oligo-dT poly(A) selection methods [61], using a T-window size of 20 bp (equivalent to the primer T sequence) and removed. Read annotation was performed with TALON, using the human Gencode v.29 reference annotation gtf with minimum alignment identity = 0.9 and coverage = 0.8. All 10 cDNA ONT replicates were utilised to maximise recovery of novel features. Identified transcripts were subsequently filtered using a minimum count threshold of *N* = 5 reads in K = 3 samples. As we expect to find lowly expressed isoforms in both biological conditions (*N* = 5 replicates per condition), K = 3 was selected to balance sensitivity with accuracy. An updated annotation was produced using this filtered set of transcripts. The TALON custom gtf contains only features detected with reads present in the dataset, so a complete custom transcriptome annotation was compiled by merging the reference and TALON gtfs.

We then employed a series of quality control and validation steps. Novel antisense transcripts perfectly matching existing gene models were removed. Novel features were then validated by assessing short read exon coverage with bedtools v.2.28.0 [62] using the orthogonal cDNA short read data genome-aligned with HISAT v.2.0.5 [63] and a minimum threshold of 15 reads depth over at least 75% of exon length. Any entries containing novel exons that failed this validation were removed from the gtf. The resulting validated annotation file is herein referred to as the TALON gtf (Fig. S1 for full pipeline). Novel features were explored utilising a series of custom scripts to assess frameshifting and coding potential with CPAT v.2.0 [36] and publicly available data within the UCSC browser and associated databases [64]. The subset of novel transcripts containing putative novel transcription start sites were identified by comparison with known start sites in the GTF and further validated by assessing overlap with Fantom5 CAGE peak data [37]. A window between the putative novel start site and 500 bp upstream was calculated for each transcript, converted from hg38 to hg19 using UCSC liftover (http://genome.ucsc.edu/cgi-bin/hgLiftOver) [64] and intersected against the hg19 phase1 & 2 combined coordinated peak data (https://fantom.gsc.riken.jp/5/datafiles/latest/extra/CAGE_peaks/hg19.cage_peak_phase1and2combined_coord.bed.gz) using bedtools.

The *CACNA2D2* novel exon was validated by RT-PCR reaction targeting the novel exon, novel junction and previously-described exonic fragment (see supplementary materials for details). Additional validation was obtained by assessing orthogonal short read coverage of all 10 samples using bedtools coverage and thirdly by investigation of human cortex RNA-seq data by accessing 27 accessions from 21 individuals in the publicly available GTEx database in the same manner.

### Differential expression analyses

#### Sequin spike-in detection & ONT DE sensitivity

Sensitivity in detecting isoform DE using ONT was assessed by a) finding the threshold of detection for each Sequin mix, b) comparing observed vs expected logFC and c) comparing with short read data. Read TPM (transcripts per million) was calculated with Salmon v.0.13.1 [65] and analysed with Anaquin v.2.8.0 [66]. Anaquin finds the limit of quantification (LOQ) concentration for each Sequin mix by fitting linear regression on the entire dataset, minimising the total sum of squares of differences between variables [66]. This was also performed for both the full short read data and a version downsampled to equivalent ONT average nucleotide coverage using bedtools. Differential expression analyses were performed with edgeR v.3.30.3 [67] in R v.4.0.2 [68]. Counts (numReads) were generated from unaligned reads at transcript-level using Salmon in mapping-based mode for the Sequins in each replicate. We then utilised a standard differential expression pipeline (detailed below). The differential expression regression model was specified by splitting the data into Sequin MixA and MixB accordingly. Expected log-fold change (logFC) was calculated as log_2_(MixB/MixA-1) + 1 for each Sequin spike-in, for direct comparison with the observed logFC calculated with edgeR.

#### Gene and transcript-level differential expression

Differential expression analyses were carried out at both transcript (DTE) and gene (DGE) level. ONT reads were mapped to the TALON transcriptome using minimap2 and quantified with Salmon in alignment-based mode and using 100 bootstrap replicates. Any novel transcripts from the Talon custom annotation that were not located on assembled chromosomes were removed to reduce any impact from counting errors associated with scaffold-only/duplicated transcripts. Transcript-level counts were then obtained by importing Salmon results with the edgeR function catchSalmon(), using the bootstrap replicates to calculate and apply an overdispersion correction for each count. As ONT reads achieve relatively low coverage compared with short reads, any transcripts unexpressed/undetected across all the 10 samples were removed from the data but all other counts were retained. Gene-level counts were generated by subsequently removing these unexpressed/undetected transcripts from the Salmon quantification (quant.sf) files and importing these pre-filtered data directly to edgeR with tximport v.1.16.1 [69] and an isoform-to-gene conversion matrix built from the TALON gtf. We then utilised a standard differential expression pipeline in edgeR for both DTE and DGE. The data were TMM-normalised and the biological coefficient of variation (BCV) and multidimensional scaling (MDS) were both manually assessed for outliers and confounding variation in the dataset. In each analysis, a model matrix was specified and applied to a glm, with false discovery rate (Benjamini-Hochberg FDR) correction for multiple testing of the results. Both DTE and DGE results were also filtered at a threshold of ±1.5 logFC (log2 fold change) and FDR < 0.05, to obtain the most differentially expressed subset of features in each case for downstream analyses.

#### Differential usage analyses

Differential transcript usage (DTU) was assessed using the R package IsoformSwitchAnalyzeR v.1.11.3 [70] on the same transcript quantification input used for DTE and DGE. TPM abundances were imported using the scaledTPM function in tximport and imported into IsoformSwitchAnalyzeR. The DTU analysis was run in two parts; first non-expressed isoforms were removed, and switches calculated for each gene using DEXseq [71] and nucleotide and peptide outputs for each gene were created for protein assessment. Transcripts were assessed for coding potential with CPAT [36], protein domain assignment with PFam [72], signal peptide prediction with SignalP v.5.0 [52] and intrinsically disordered regions and binding regions with IUPred2A [73], using default parameters according to the IsoformSwitchAnalyzeR workflow. The second part of the IsoformSwitchAnalyzeR DTU analysis then leveraged these data to identify isoforms switches with potential functional consequences and provide visualisation using default functions. IsoformSwitchAnalyzeR was also used to provide a genome-wide overview of the number alternative splicing events (alternative donor and acceptor sites, intron retention, alternative first and last exons, mutually exclusive exons and exon skipping) skipping during the differentiation process.

#### Ontology and functional association

To interpret the differentially expressed or used gene sets, we assessed gene ontology and known associations with neurologically relevant biology. We used the GENE2FUNC function in FUMA (functional mapping and annotation, https://fuma.ctglab.nl/) [74] to annotate the gene sets within a biological context. For transcripts, the corresponding Ensembl gene ID was used. In each case, the default thresholds of significance and ontology enrichment were applied. Analyses focused on tissue specificity analyses in GTEx v.8 30 tissue types and Gene Ontogeny (GO) Biological Processes.

## Supplementary Information

**Additional file 1: Figure S1.** Schematic representation of the custom annotation pipeline, utilising TALON software (Wyman et al. 2020) with custom bash, python and perl auxiliary and processing scripts (collated in clean_TALON_output.pl, see script repository). **Fig. S2.** Schematic representation of *CACNA2D2* (ENSG00000007402) transcripts, showing the novel transcript TALONT000703030. Figure modified from IsoformSwitchAnalyzeR output (Vitting-Seerup and Sandelin 2019). **Fig. S3.** Short read (Illumina paired-end) coverage plot of novel first exon (31 bp) of CACNA2D2 (ENSG00000007402) transcript TALONT000703030 from all 10 sequencing runs (see Table S1). **Fig. S4.** Coverage plot of novel first exon (31 bp) of CACNA2D2 (ENSG00000007402) transcript TALONT000703030 from *N* = 27 human cortex RNA-seq GTEx accessions from *N* = 21 individuals: SRR1310008, SRR1311400, SRR1311575, SRR1315866, SRR1316815, SRR1317344, SRR1320963, SRR1323043, SRR1326179, SRR1331579, SRR1333930, SRR1337564, SRR1339651, SRR1343481, SRR1353176, SRR1354446, SRR1364676, SRR1368772, SRR1382732, SRR1383059, SRR1387809, SRR1418837, SRR1418992, SRR1433971, SRR1435293, SRR1444580, SRR1468514. **Fig. S5.** Comparison of an example annotated coding transcript (ENST00000479441) of *CACNA2D2* (ENSG00000007402) with the novel transcript TALONT000703030, demonstrating key differences and initial 3D structure rendered using Phyre2 (Kelley et al. 2015). **Fig. S6.** Schematic representation of primer placement for RT-PCR validation of the novel *CACNA2D2* exon and transcript (TALON000703030) relative to two representative examples of previously known transcripts. Note, the same reverse primer is used for each forward. See Table S5 for primer details. **Fig. S7.** Gel electrophoresis image of the *CACNA2D2* RT-PCR validation. Each primer set is labelled corresponding to Table S5 and Fig S6, along with the negative and positive controls. **Fig. S8.** Custom UCSC Genome Browser visualization of the full coverage of short read (pink) and long read RNA-Seq (blue) across the *CACNA2D2* genome model for a single sample (differentiated cells; sample D2). Read peaks supporting the novel exon shown on far right of tracks. **Table S1.** Summary statistics of the Oxford Nanopore Technologies (ONT) after quality checking and Illumina paired-end short read sequencing (SRS) of 10 replicate samples of human neuronal cell line SH-SY5Y. D = differentiated cell and U = undifferentiated cell samples. **Table S2.** Comparison of limit of quantification (LOQ) of Oxford Nanopore Technologies (ONT) sequencing, Illumina short read sequencing (SRS) and Illumina reads downsampled to average nucleotide coverage of ONT reads. LOQ calculated by Anaquin (Wong et al. 2017). **Table S3.** Differential gene and transcript expression at a stringent filter of logFC ⋛ 1.5, FDR q-value < 0.05 (see also Fig. 3A and C). Bracketed numbers refer to the portion of total that are TALON-identified novel transcripts. U = undifferentiated and D = differentiated cells, with arrows displaying expression directionality. **Table S4.** *N* = 104 Differential transcript usage switches with functional consequence ranked by q-value. Output from IsoformSwitchAnalyzeR (Vitting-Seerup and Sandelin 2019). Vitting-Seerup et al define the gene dIF values as the total change within the gene calculated as sum (abs(dIF)) of the transcripts. **Table S5.** *CACNA2D2* RT-PCR validation primers designed with Primer-BLAST (Ye et al. 2012). All primer sets used the same reverse primer. SC = self-complementarity and S3’C = Self 3′ complementarity. **Table S6.** List of *N* = 333 novel transcripts possessing putatively novel transcription start sites and which display CAGEseq peak overlap (± 500 bp). Chromosome, overlap interval start and end and novel TALON transcript ID are provided. **Table S7.** Overview of genome-wide alternative splicing events between cell states during differentiation of SHSY5Y cells. IF = Isoform Fraction. 1 = differentiated, 2 = undifferentiated. Each row is a comparison between differentiated vs undifferentiated cells. A5 & A3 = alternative donor and acceptor sites, IR = intron retention, ATSS = alternative first exon, ATTS = alternative last exon, MEE = mutually exclusive exon, ES = exon skipping, MES = multiple exon skipping. Produced with IsoformSwitchAnalyzeR.

## Data Availability

The datasets supporting the conclusions of this article are available in the following repositories: read data are archived in ENA (short reads: PRJEB44501, long reads: PRJEB44502). Analysis scripts are available from https://github.com/TGAC/SHSY5Y_differentiation and the accompanying datafiles from 10.5281/zenodo.4727856 or the corresponding author.

## Abbreviations

AS: Alternative splicing
DE: Differential expression
DGE: Differential gene expression
DTE: Differential transcript expression
DTU: Differential transcript usage
FDR: False discovery rate
logFC: Log_2_ fold-change
ONT: Oxford Nanopore Technologies
RBPs: RNA-binding proteins
VGCC: Voltage gated calcium channel

## References

[1] Breschi A, Muñoz-Aguirre M, Wucher V, Davis CA, Garrido-Martín D, Djebali S (2020). A limited set of transcriptional programs define major cell types. Genome Res.

[2] Chepelev I, Chen X (2013). Alternative splicing switching in stem cell lineages. Front Biol.

[3] Grabowski P (2011). Alternative splicing takes shape during neuronal development. Curr Opin Genet Dev.

[4] Ule J, Ule A, Spencer J, Williams A, Hu J-S, Cline M (2005). Nova regulates brain-specific splicing to shape the synapse. Nat Genet.

[5] Raj B, Blencowe BJ (2015). Alternative splicing in the mammalian nervous system: recent insights into mechanisms and functional roles. Neuron..

[6] Weyn-Vanhentenryck SM, Feng H, Ustianenko D, Duffié R, Yan Q, Jacko M (2018). Precise temporal regulation of alternative splicing during neural development. Nat Commun.

[7] Liu J, Geng A, Wu X, Lin R-J, Lu Q (2018). Alternative RNA splicing associated with mammalian neuronal differentiation. Cereb Cortex.

[8] Burke EE, Chenoweth JG, Shin JH, Collado-Torres L, Kim S-K, Micali N (2020). Dissecting transcriptomic signatures of neuronal differentiation and maturation using iPSCs. Nat Commun.

[9] Saito Y, Yuan Y, Zucker-Scharff I, Fak JJ, Jereb S, Tajima Y (2019). Differential NOVA2-mediated splicing in excitatory and inhibitory neurons regulates cortical development and cerebellar function. Neuron..

[10] Boutz PL, Stoilov P, Li Q, Lin C-H, Chawla G, Ostrow K (2007). A post-transcriptional regulatory switch in polypyrimidine tract-binding proteins reprograms alternative splicing in developing neurons. Genes Dev.

[11] Linares AJ, Lin C-H, Damianov A, Adams KL, Novitch BG, Black DL (2015). The splicing regulator PTBP1 controls the activity of the transcription factor Pbx1 during neuronal differentiation. Elife..

[12] Keppetipola N, Sharma S, Li Q, Black DL (2012). Neuronal regulation of pre-mRNA splicing by polypyrimidine tract binding proteins, PTBP1 and PTBP2. Crit Rev Biochem Mol Biol.

[13] Jackson TC, Janesko-Feldman K, Gorse K, Vagni VA, Jackson EK, Kochanek PM (2020). Identification of novel targets of RBM5 in the healthy and injured brain. Neuroscience..

[14] Gallego-Paez LM, Bordone MC, Leote AC, Saraiva-Agostinho N, Ascensão-Ferreira M, Barbosa-Morais NL (2017). Alternative splicing: the pledge, the turn, and the prestige : the key role of alternative splicing in human biological systems. Hum Genet.

[15] Clark TA, Schweitzer AC, Chen TX, Staples MK, Lu G, Wang H (2007). Discovery of tissue-specific exons using comprehensive human exon microarrays. Genome Biol.

[16] Yi L, Pimentel H, Bray NL, Pachter L (2018). Gene-level differential analysis at transcript-level resolution. Genome Biol.

[17] Yuste R, Hawrylycz M, Aalling N, Aguilar-Valles A, Arendt D, Arnedillo RA, et al. A community-based transcriptomics classification and nomenclature of neocortical cell types. Nat Neurosci. 2020. 10.1038/s41593-020-0685-8. 10.1038/s41593-020-0685-8 PMC7683348 32839617

[18] Clark MB, Wrzesinski T, Garcia AB, Hall NAL, Kleinman JE, Hyde T (2020). Long-read sequencing reveals the complex splicing profile of the psychiatric risk gene CACNA1C in human brain. Mol Psychiatry.

[19] Scotti MM, Swanson MS (2016). RNA mis-splicing in disease. Nat Rev Genet.

[20] Jaudon F, Baldassari S, Musante I, Thalhammer A, Zara F, Cingolani LA. Targeting alternative splicing as a potential therapy for episodic Ataxia type 2. Biomedicines. 2020;8. 10.3390/biomedicines8090332. 10.3390/biomedicines8090332 PMC7555146 32899500

[21] Splawski I, Timothy KW, Sharpe LM, Decher N, Kumar P, Bloise R (2004). Ca(V)1.2 calcium channel dysfunction causes a multisystem disorder including arrhythmia and autism. Cell..

[22] Gandal MJ, Zhang P, Hadjimichael E, Walker RL, Chen C, Liu S, et al. Transcriptome-wide isoform-level dysregulation in ASD, schizophrenia, and bipolar disorder. Science. 2018;362. 10.1126/science.aat8127. 10.1126/science.aat8127 PMC6443102 30545856

[23] Stark R, Grzelak M, Hadfield J (2019). RNA sequencing: the teenage years. Nat Rev Genet.

[24] Wang Z, Gerstein M, Snyder M (2009). RNA-Seq: a revolutionary tool for transcriptomics. Nat Rev Genet.

[25] Byrne A, Beaudin AE, Olsen HE, Jain M, Cole C, Palmer T (2017). Nanopore long-read RNAseq reveals widespread transcriptional variation among the surface receptors of individual B cells. Nat Commun.

[26] Byrne A, Cole C, Volden R, Vollmers C (2019). Realizing the potential of full-length transcriptome sequencing. Philos Trans R Soc Lond Ser B Biol Sci.

[27] Wang X, You X, Langer JD, Hou J, Rupprecht F, Vlatkovic I (2019). Full-length transcriptome reconstruction reveals a large diversity of RNA and protein isoforms in rat hippocampus. Nat Commun.

[28] Sessegolo C, Cruaud C, Da Silva C, Cologne A, Dubarry M, Derrien T (2019). Transcriptome profiling of mouse samples using nanopore sequencing of cDNA and RNA molecules. Sci Rep.

[29] Kovalevich J, Langford D (2013). Considerations for the use of SH-SY5Y neuroblastoma cells in neurobiology. Methods Mol Biol.

[30] Shipley MM, Mangold CA, Szpara ML. Differentiation of the SH-SY5Y human neuroblastoma cell line. J Vis Exp. 2016;108:53193. 10.3791/53193 PMC4828168 26967710

[31] Agholme L, Lindström T, Kågedal K, Marcusson J, Hallbeck M (2010). An in vitro model for neuroscience: differentiation of SH-SY5Y cells into cells with morphological and biochemical characteristics of mature neurons. J Alzheimers Dis.

[32] Truckenmiller ME, Vawter MP, Cheadle C, Coggiano M, Donovan DM, Freed WJ (2001). Gene expression profile in early stage of retinoic acid-induced differentiation of human SH-SY5Y neuroblastoma cells. Restor Neurol Neurosci.

[33] Forster JI, Köglsberger S, Trefois C, Boyd O, Baumuratov AS, Buck L (2016). Characterization of differentiated SH-SY5Y as neuronal screening model reveals increased oxidative vulnerability. J Biomol Screen.

[34] Mendsaikhan A, Takeuchi S, Walker DG, Tooyama I (2018). Differences in gene expression profiles and phenotypes of differentiated SH-SY5Y neurons stably overexpressing mitochondrial ferritin. Front Mol Neurosci.

[35] Hardwick SA, Chen WY, Wong T, Deveson IW, Blackburn J, Andersen SB (2016). Spliced synthetic genes as internal controls in RNA sequencing experiments. Nat Methods.

[36] Wang L, Park HJ, Dasari S, Wang S, Kocher J-P, Li W (2013). CPAT: coding-potential assessment tool using an alignment-free logistic regression model. Nucleic Acids Res.

[37] Lizio M, Abugessaisa I, Noguchi S, Kondo A, Hasegawa A, Hon CC (2019). Update of the FANTOM web resource: expansion to provide additional transcriptome atlases. Nucleic Acids Res.

[38] Carithers LJ, Ardlie K, Barcus M, Branton PA, Britton A, Buia SA (2015). A novel approach to high-quality postmortem tissue procurement: the GTEx project. Biopreserv Biobank.

[39] Warga RM, Wicklund A, Webster SE, Kane DA (2016). Progressive loss of RacGAP1/ogre activity has sequential effects on cytokinesis and zebrafish development. Dev Biol.

[40] Jackson TC, Kochanek PM (2020). RNA binding motif 5 (RBM5) in the CNS-moving beyond Cancer to harness RNA splicing to mitigate the consequences of brain injury. Front Mol Neurosci.

[41] Glinos DA, Garborcauskas G, Hoffman P, Ehsan N, Jiang L, Gokden A, et al. Transcriptome variation in human tissues revealed by long-read sequencing. bioRxiv. 2021;:2021.01.22.427687. 10.1101/2021.01.22.427687. 10.1038/s41586-022-05035-y PMC10337767 35922509

[42] Gleeson J, Lane TA, Harrison PJ, Haerty W, Clark MB. Nanopore direct RNA sequencing detects differential expression between human cell populations. Cold Spring Harbor Lab. 2020;:2020.08.02.232785. 10.1101/2020.08.02.232785.

[43] Soneson C, Yao Y, Bratus-Neuenschwander A, Patrignani A, Robinson MD, Hussain S (2019). A comprehensive examination of Nanopore native RNA sequencing for characterization of complex transcriptomes. Nat Commun.

[44] Conn KJ, Ullman MD, Larned MJ, Eisenhauer PB, Fine RE, Wells JM (2003). cDNA microarray analysis of changes in gene expression associated with MPP+ toxicity in SH-SY5Y cells. Neurochem Res.

[45] Dolphin AC (2016). Voltage-gated calcium channels and their auxiliary subunits: physiology and pathophysiology and pharmacology. J Physiol.

[46] Gao B, Sekido Y, Maximov A, Saad M, Forgacs E, Latif F (2000). Functional properties of a new voltage-dependent calcium channel alpha(2)delta auxiliary subunit gene (CACNA2D2). J Biol Chem.

[47] Brill J, Klocke R, Paul D, Boison D, Gouder N, Klugbauer N (2004). Entla, a novel epileptic and ataxic Cacna2d2 mutant of the mouse. J Biol Chem.

[48] Barclay J, Balaguero N, Mione M, Ackerman SL, Letts VA, Brodbeck J (2001). Ducky mouse phenotype of epilepsy and ataxia is associated with mutations in the Cacna2d2 gene and decreased calcium channel current in cerebellar Purkinje cells. J Neurosci.

[49] Brodbeck J, Davies A, Courtney J-M, Meir A, Balaguero N, Canti C (2002). The ducky mutation in Cacna2d2 results in altered Purkinje cell morphology and is associated with the expression of a truncated alpha 2 delta-2 protein with abnormal function. J Biol Chem.

[50] Kelley LA, Mezulis S, Yates CM, Wass MN, Sternberg MJE (2015). The Phyre2 web portal for protein modeling, prediction and analysis. Nat Protoc.

[51] Hofmann F, Flockerzi V, Kahl S, Wegener JW (2014). L-type CaV1.2 calcium channels: from in vitro findings to in vivo function. Physiol Rev.

[52] Armenteros JJA, Tsirigos KD, Sønderby CK, Petersen TN, Winther O, Brunak S (2019). SignalP 5.0 improves signal peptide predictions using deep neural networks. Nat Biotechnol.

[53] Dolphin AC. Voltage-gated calcium channel α 2δ subunits: an assessment of proposed novel roles. F1000Res. 2018;7. 10.12688/f1000research.16104.1. 10.12688/f1000research.16104.1 PMC6249638 30519455

[54] Ewels P, Magnusson M, Lundin S, Käller M (2016). MultiQC: summarize analysis results for multiple tools and samples in a single report. Bioinformatics..

[55] Krueger F (2015). Trim galore.

[56] Broad Institute (2019). Picard toolkit.

[57] Li H (2018). Minimap2: pairwise alignment for nucleotide sequences. Bioinformatics..

[58] Wyman D, Balderrama-Gutierrez G, Reese F, Jiang S, Rahmanian S, Forner S, et al. A technology-agnostic long-read analysis pipeline for transcriptome discovery and quantification. bioRxiv. 2020:672931. 10.1101/672931.

[59] Wyman D, Mortazavi A (2019). TranscriptClean: variant-aware correction of indels, mismatches and splice junctions in long-read transcripts. Bioinformatics..

[60] Sibley CR, Blazquez L, Ule J (2016). Lessons from non-canonical splicing. Nat Rev Genet.

[61] Roy KR, Chanfreau GF (2020). Robust mapping of polyadenylated and non-polyadenylated RNA 3′ ends at nucleotide resolution by 3′-end sequencing. Methods..

[62] Quinlan AR, Hall IM (2010). BEDTools: a flexible suite of utilities for comparing genomic features. Bioinformatics..

[63] Kim D, Langmead B, Salzberg SL (2015). HISAT: a fast spliced aligner with low memory requirements. Nat Methods.

[64] Kent WJ, Sugnet CW, Furey TS, Roskin KM, Pringle TH, Zahler AM (2002). The human genome browser at UCSC. Genome Res.

[65] Patro R, Duggal G, Love MI, Irizarry RA, Kingsford C (2017). Salmon provides fast and bias-aware quantification of transcript expression. Nat Methods.

[66] Wong T, Deveson IW, Hardwick SA, Mercer TR (2017). ANAQUIN: a software toolkit for the analysis of spike-in controls for next generation sequencing. Bioinformatics..

[67] McCarthy DJ, Chen Y, Smyth GK (2012). Differential expression analysis of multifactor RNA-Seq experiments with respect to biological variation. Nucleic Acids Res.

[68] R Core Team. R: A language and environment for statistical computing. R Foundation for Statistical Computing, Vienna, Austria. 2021. https://www.R-project.org/.

[69] Soneson C, Love MI, Robinson MD (2015). Differential analyses for RNA-seq: transcript-level estimates improve gene-level inferences. F1000Res.

[70] Vitting-Seerup K, Sandelin A (2019). IsoformSwitchAnalyzeR: analysis of changes in genome-wide patterns of alternative splicing and its functional consequences. Bioinformatics..

[71] Anders S, Reyes A, Huber W (2012). Detecting differential usage of exons from RNA-seq data. Genome Res.

[72] Punta M, Coggill PC, Eberhardt RY, Mistry J, Tate J, Boursnell C (2012). The Pfam protein families database. Nucleic Acids Res.

[73] Mészáros B, Erdos G, Dosztányi Z (2018). IUPred2A: context-dependent prediction of protein disorder as a function of redox state and protein binding. Nucleic Acids Res.

[74] Watanabe K, Taskesen E, van Bochoven A, Posthuma D (2017). Functional mapping and annotation of genetic associations with FUMA. Nat Commun.

