# Correction to: Long read sequencing reveals novel isoforms and insights into splicing regulation during cell state changes

**DOI:** 10.1186/s12864-022-08318-w

**Published:** 2022-01-25

**Authors:** David J. Wright, Nicola A. L. Hall, Naomi Irish, Angela L. Man, Will Glynn, Arne Mould, Alejandro De Los Angeles, Emily Angiolini, David Swarbreck, Karim Gharbi, Elizabeth M. Tunbridge, Wilfried Haerty

**Affiliations:** 1 Earlham Institute, Norwich Research Park, Norfolk, NR4 7UZ UK; 2 Department of Psychiatry, Medical Sciences Division, University of Oxford, Oxfordshire, OX3 3JX UK; 3 Oxford Health, NHS Foundation Trust, Oxford, Oxfordshire OX3 7JX UK

**Correction to: BMC Genomics 23, 42 (2022)**

**https://doi.org/10.1186/s12864-021-08261-2**

Following publication of the original article [1], it was that there was an error in Fig. 4. There was an overlaid panel in the center of the image. The correct Fig. 4 is provided in this Correction and the original article [1] has been updated.

**Fig. 4:**
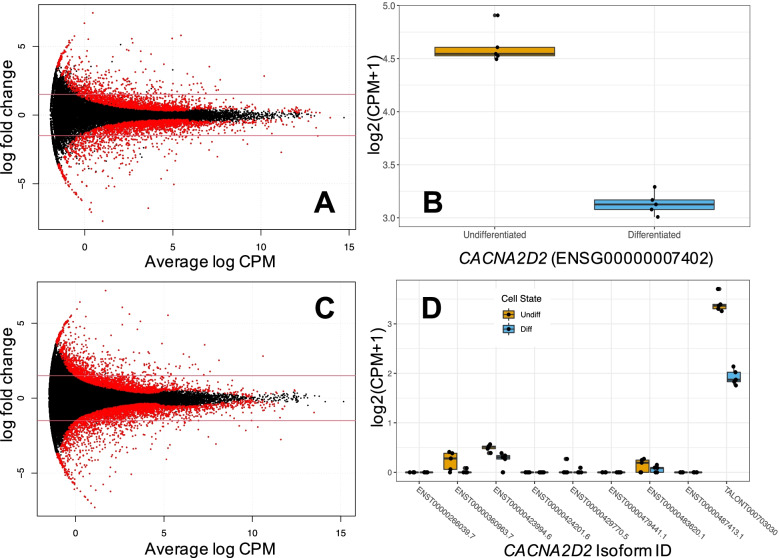
Panel of **A** gene-level differential expression (DE) smear plot, solid red lines highlighting ±1.5 logFC threshold, **B** gene-level DE of *CACNA2D2* during differentiation, **C** isoform-level DE smear plot with ±1.5 logFC threshold, **D** *CACNA2D2* isoform expression, showing novel TALON isoform with highest read count. Red points on smear plots indicate significant differential expression (FDR < 0.05). Boxplots display median and IQR. Short and long read mapping example provided in Fig. S8
