# Supplementary material for: Long read sequencing reveals novel isoforms and insights into splicing regulation during cell state changes
Source: BMC Genomics. 2022 Jan 10;23:42. doi: 10.1186/s12864-021-08261-2 (PMC8744310; doi:10.1186/s12864-021-08261-2)
Supplement: Supplementary file 1 — Additional file 1: Figure S1. Schematic representation of the custom annotation pipeline, utilising TALON software (Wyman et al. 2020) with custom bash, python and perl auxiliary and processing scripts (collated in clean_TALON_output.pl, see script repository). Fig. S2. Schematic representation of CACNA2D2 (ENSG00000007402) transcripts, showing the novel transcript TALONT000703030. Figure modified from IsoformSwitchAnalyzeR output (Vitting-Seerup and Sandelin 2019). Fig. S3. Short read (Illumina paired-end) coverage plot of novel first exon (31 bp) of CACNA2D2 (ENSG00000007402) transcript TALONT000703030 from all 10 sequencing runs (see Table S1). Fig. S4. Coverage plot of novel first exon (31 bp) of CACNA2D2 (ENSG00000007402) transcript TALONT000703030 from N = 27 human cortex RNA-seq GTEx accessions from N = 21 individuals: SRR1310008, SRR1311400, SRR1311575, SRR1315866, SRR1316815, SRR1317344, SRR1320963, SRR1323043, SRR1326179, SRR1331579, SRR1333930, SRR1337564, SRR1339651, SRR1343481, SRR1353176, SRR1354446, SRR1364676, SRR1368772, SRR1382732, SRR1383059, SRR1387809, SRR1418837, SRR1418992, SRR1433971, SRR1435293, SRR1444580, SRR1468514. Fig. S5. Comparison of an example annotated coding transcript (ENST00000479441) of CACNA2D2 (ENSG00000007402) with the novel transcript TALONT000703030, demonstrating key differences and initial 3D structure rendered using Phyre2 (Kelley et al. 2015). Fig. S6. Schematic representation of primer placement for RT-PCR validation of the novel CACNA2D2 exon and transcript (TALON000703030) relative to two representative examples of previously known transcripts. Note, the same reverse primer is used for each forward. See Table S5 for primer details. Fig. S7. Gel electrophoresis image of the CACNA2D2 RT-PCR validation. Each primer set is labelled corresponding to Table S5 and Fig S6, along with the negative and positive controls. Fig. S8. Custom UCSC Genome Browser visualization of the full coverage of short read (pink) and long read [file 12864_2021_8261_MOESM1_ESM.docx]

**Supplementary Information: Long read sequencing reveals novel isoforms and insights into splicing regulation during neuron-like cell differentiation**

David J Wright^1^, Naomi Irish^1^, Nicola Hall^2,3^, Angela Man^1^, Will Glynn^1^, Arne Mould^2,3^, Alejandro De Los Angeles^2,3^, Emily Angiolini^1^, David Swarbreck^1^, Karim Gharbi^1^, Elizabeth Tunbridge^2,3^, Wilfried Haerty^1^*

^1^Earlham Institute, Norwich Research Park, Norfolk NR5 0PB, UK

^2^Department of Psychiatry, Medical Sciences Division, University of Oxford, Oxfordshire OX3 3JX, UK

^3^National Institute for Health Research, Oxford Health Biomedical Research Centre, Oxfordshire OX3 7JX, UK

* corresponding author: Wilfried.Haerty@earlham.ac.uk

**Supplementary Results & Discussion**

***Gene & transcript-level differential expression at more stringent log fold change thresholds***

Using more stringent threshold criteria (logFC ⪌ 1.5), 503 genes were upregulated in the differentiated cells, and 524 downregulated (Table S3), compared to the undifferentiated state (Fig 3A). These upregulated genes also showed greatest overlap with those up-regulated in the brain, compared with other tissue types (p_adj_= 1.2 x 10^-23^). Whilst the permissive data showed significant overlap with genes down-regulated in the brain, this relationship was not observed at the more stringent threshold (p_adj_= 1).

At the transcript-level, we found a total of 884 transcripts significantly upregulated and 875 significantly downregulated in the differentiated cells (Fig 3C). These include 28 upregulated and 21 downregulated novel TALON transcripts respectively (Table S3).

**Supplementary Methods**

***CACNA2D2 novel exon / transcript validation***

Laboratory validation of the novel *CACNA2D2* exon/transcript was conducted with 3 RT-PCR primer sets designed using Primer-BLAST [(Ye et al. 2012)](https://paperpile.com/c/3gOEAj/MncP0). Forward primers were situated in the novel exon, spanning the novel junction and in the upstream previously known exons with a single reverse primer utilised in all 3 sets (Figs. S6, S7, Table S5). Primers were synthesised by Integrated DNA Technologies. Total RNA extracted from the 10 replicate human neuroblastoma SH-SY5Y cell lines treated with (D1-D5) or without (U1-U5) differentiating media, as described in the Materials and Methods, were used as template for the RT-PCR.

5 µg of total RNA was treated with Invitrogen™ ezDNase™ Enzyme and first strand cDNA was synthesised from this RNA using Invitrogen™ SuperScript™ IV First-Strand Synthesis System (Life Technologies) using 50uM Oligo d(T)20 in 20 µl volumes following the manufacturer’s protocol. All samples were treated with *E. coli* RNase H.

First strand cDNA was then used as template in PCR amplification using Q5® High-Fidelity 2X Master Mix (New England Biolabs) following the manufacturer’s protocol for 50 µl volume reactions with annealing temperature/time of 65 °C and 30 sec, respectively. The products were then visualised with electrophoresis using SYBR™ Safe DNA Stain (Thermofisher), 2% (w/v) agarose gel and 10 ul product loaded in each well (Fig S8).

**Supplementary Figures and Tables**


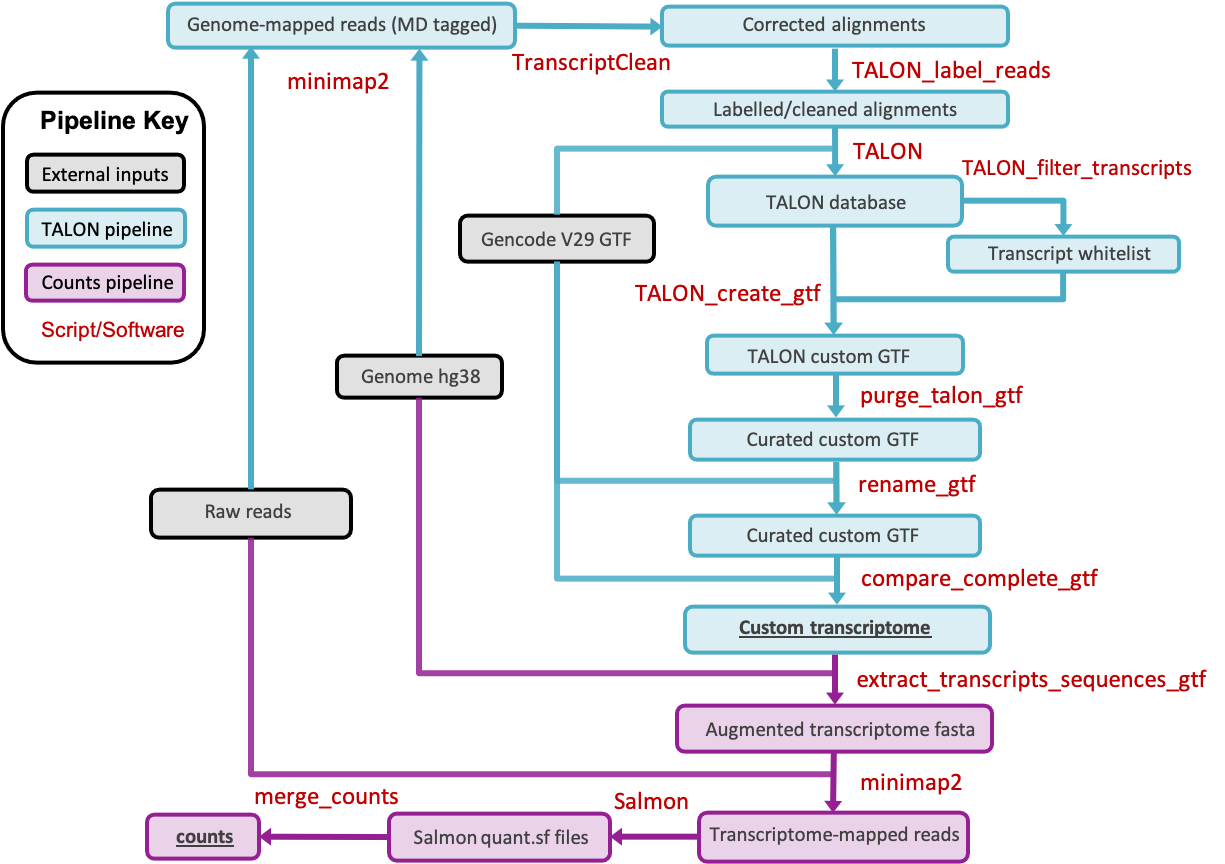


**Figure S1** Schematic representation of the custom annotation pipeline, utilising TALON software [(Wyman et al. 2020)](https://paperpile.com/c/3gOEAj/W091i) with custom bash, python and perl auxiliary and processing scripts (collated in clean_TALON_output.pl, see script repository).

**
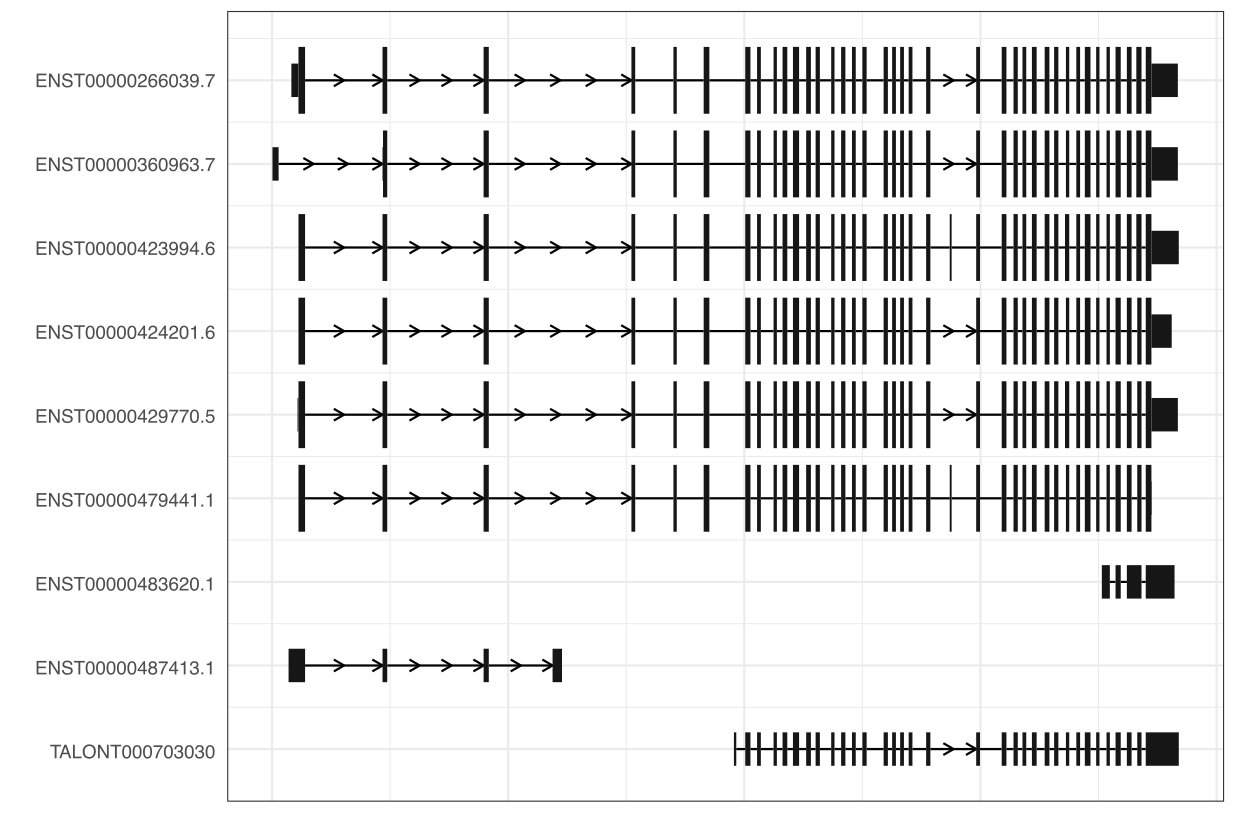
**

**Fig S2** Schematic representation of *CACNA2D2* (ENSG00000007402) transcripts, showing the novel transcript TALONT000703030. Figure modified from IsoformSwitchAnalyzeR output [(Vitting-Seerup and Sandelin 2019)](https://paperpile.com/c/3gOEAj/swFVh).

**
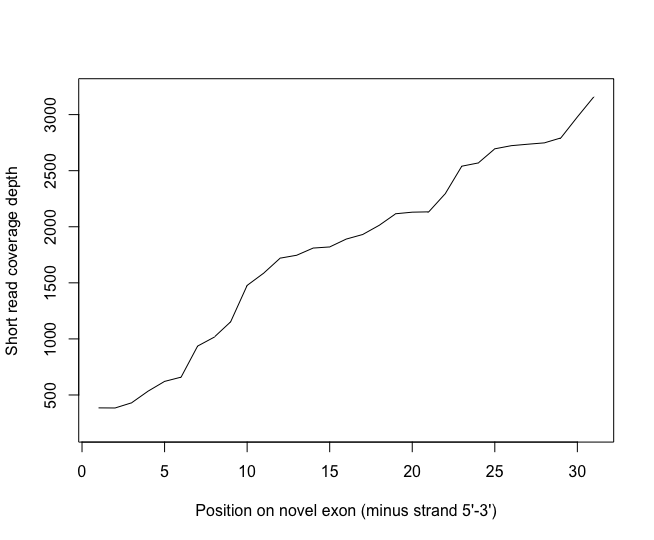
**

**Fig S3** Short read (Illumina paired-end) coverage plot of novel first exon (31bp) of CACNA2D2 (ENSG00000007402) transcript TALONT000703030 from all 10 sequencing runs (see Table S1).

**
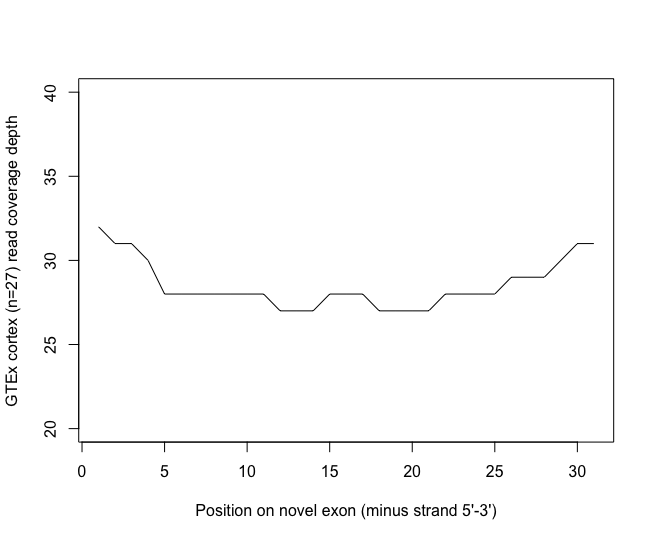
**

**Fig S4** Coverage plot of novel first exon (31bp) of CACNA2D2 (ENSG00000007402) transcript TALONT000703030 from N=27 human cortex RNA-seq GTEx accessions from N=21 individuals: SRR1310008, SRR1311400, SRR1311575, SRR1315866, SRR1316815, SRR1317344, SRR1320963, SRR1323043, SRR1326179, SRR1331579, SRR1333930, SRR1337564, SRR1339651, SRR1343481, SRR1353176, SRR1354446, SRR1364676, SRR1368772, SRR1382732, SRR1383059, SRR1387809, SRR1418837, SRR1418992, SRR1433971, SRR1435293, SRR1444580, SRR1468514.

**
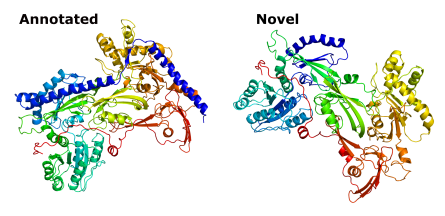
**

**Fig S5** Comparison of an example annotated coding transcript (ENST00000479441) of *CACNA2D2* (ENSG00000007402) with the novel transcript TALONT000703030, demonstrating key differences and initial 3D structure rendered using Phyre2 [(Kelley et al. 2015)](https://paperpile.com/c/3gOEAj/H86zq).


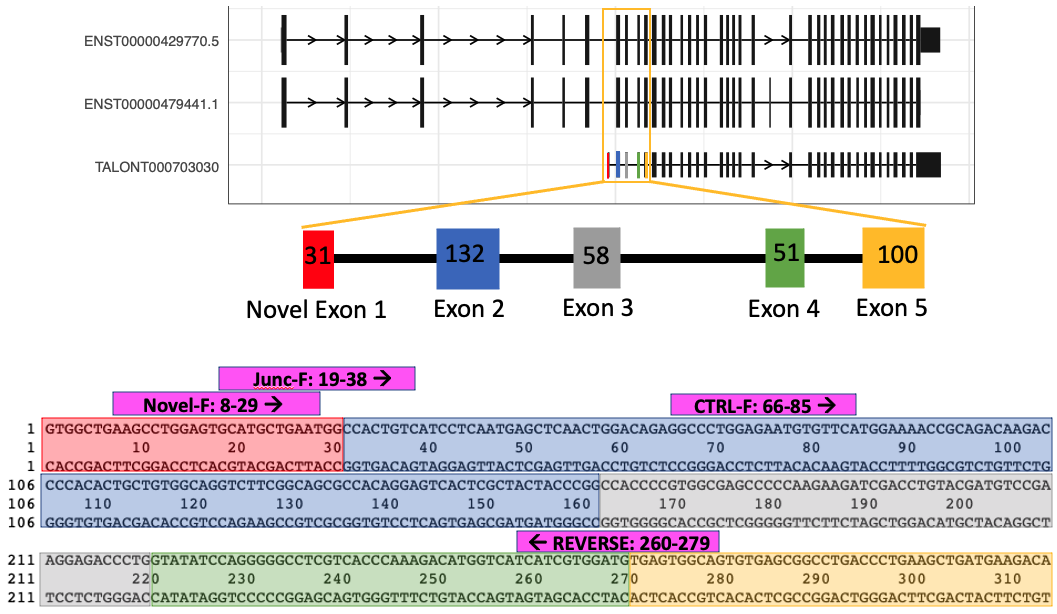


**Fig S6** Schematic representation of primer placement for RT-PCR validation of the novel *CACNA2D2* exon and transcript (TALON000703030) relative to two representative examples of previously known transcripts. Note, the same reverse primer is used for each forward. See table S5 for primer details.


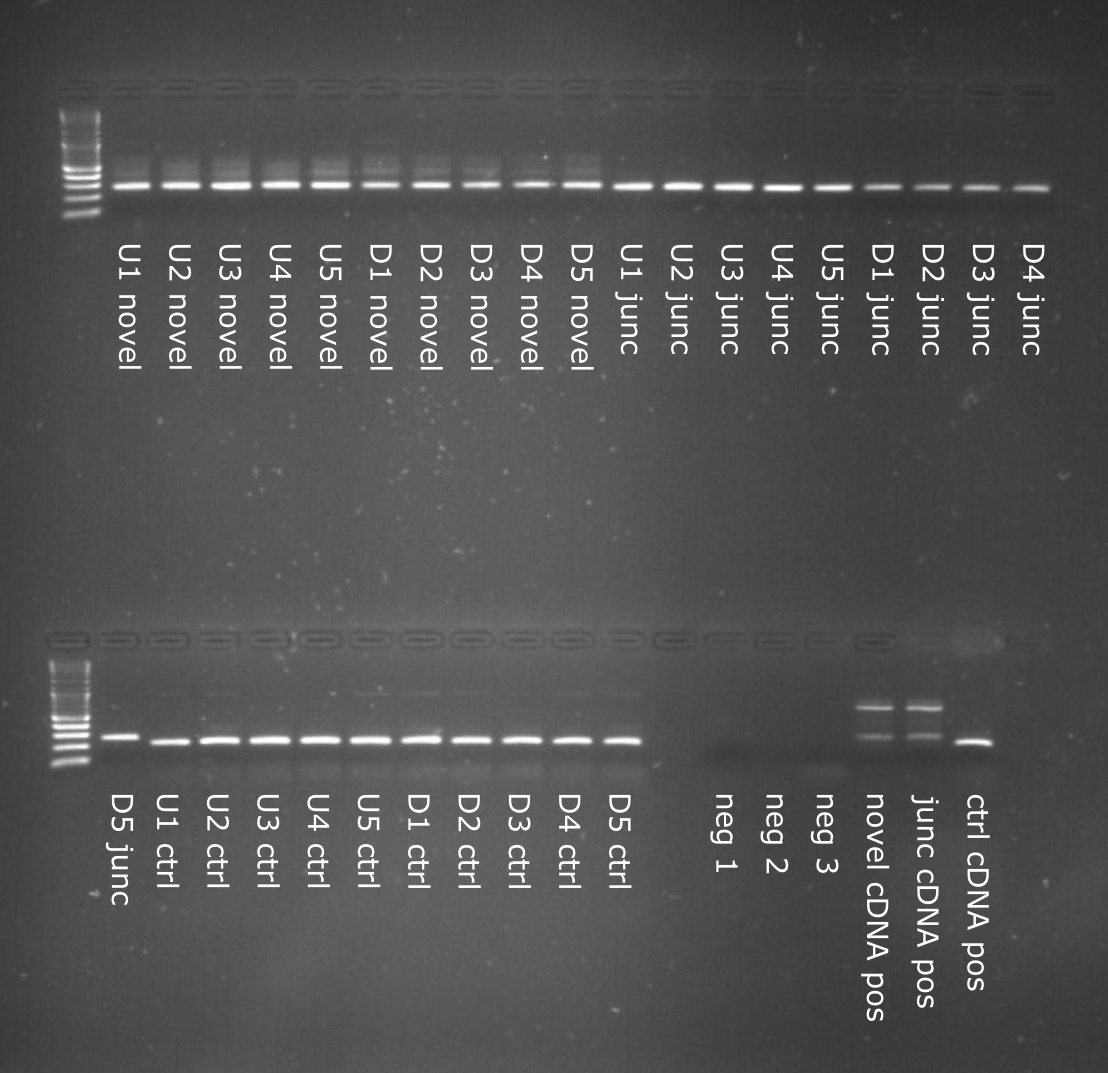


**Fig S7** Gel electrophoresis image of the *CACNA2D2* RT-PCR validation. Each primer set is labelled corresponding to Table S5 and Fig S6, along with the negative and positive controls.

**Fig S8** Custom UCSC Genome Browser visualization of the full coverage of short read (pink) and long read RNA-Seq (blue) across the *CACNA2D2* genome model for a single sample (differentiated cells; sample D2). Read peaks supporting the novel exon shown on far right of tracks.

**Table S1** Summary statistics of the Oxford Nanopore Technologies (ONT) after quality checking and Illumina paired-end short read sequencing (SRS) of 10 replicate samples of human neuronal cell line SH-SY5Y. D = differentiated cell and U = undifferentiated cell samples.

| **Sample ID** | **Reads** | **Bases** | **Median Length** | **Read N50** | **Median Quality** | **Illumina Reads** |
| --- | --- | --- | --- | --- | --- | --- |
| D1 | 11,449,796 | 9,927,703,599 | 746 | 983 | 11.53 | 109,360,175 |
| D2 | 9,792,869 | 7,733,291,052 | 675 | 951 | 11.59 | 119,690,149 |
| D3 | 11,255,431 | 9,564,769,304 | 723 | 981 | 11.53 | 116,001,216 |
| D4 | 14,236,128 | 12,584,438,699 | 732 | 1016 | 11.59 | 61,118,341 |
| D5 | 11,503,329 | 9,379,344,639 | 707 | 961 | 11.64 | 98,946,695 |
| U1 | 9,171,036 | 7,737,779,710 | 701 | 1003 | 11.49 | 109,699,478 |
| U2 | 10,753,125 | 9,199,369,288 | 723 | 975 | 11.50 | 113,071,934 |
| U3 | 7,654,475 | 6,240,728,116 | 691 | 980 | 11.61 | 120,907,938 |
| U4 | 9,805,641 | 8,331,498,610 | 720 | 989 | 11.53 | 105,973,868 |
| U5 | 11,293,547 | 9,835,274,972 | 717 | 1023 | 11.54 | 98,721,393 |

**Table S2** Comparison of limit of quantification (LOQ) of Oxford Nanopore Technologies (ONT) sequencing, Illumina short read sequencing (SRS) and Illumina reads downsampled to average nucleotide coverage of ONT reads. LOQ calculated by Anaquin [(Wong et al. 2017)](https://paperpile.com/c/3gOEAj/e1p37).

| **Sequencing Approach** | **LOQ genes (attoml/µl)** | | **LOQ isoforms (attoml/µl)** | |
| --- | --- | --- | --- | --- |
|  | **Sequin MixA** | **Sequin MixB** | **Sequin MixA** | **Sequin MixB** |
| Illumina reads (**SRS**) | - | - | - | 0.0197 |
| Downsampled Illumina | 0.118 | 0.118 | 0.059 | 0.0674 |
| Nanopore reads (**ONT**) | 0.118 | 0.472 | 0.059 | 0.270 |

**Table S3** Differential gene and transcript expression at a stringent filter of logFC ⋛ 1.5, FDR q-value < 0.05 (see also Figures 3A and 3C). Bracketed numbers refer to the portion of total that are TALON-identified novel transcripts. U = undifferentiated and D = differentiated cells, with arrows displaying expression directionality.

| Metric | Count | |
| --- | --- | --- |
|  | Gene level | Transcript level (Talon) |
| ↑U ↓︎D (≥ +1.5 logFC) | 524 | 875 (21) |
| ↑︎D ↓U (≤ -1.5 logFC) | 503 | 884 (28) |

**Table S4** N=104 Differential transcript usage switches with functional consequence ranked by q-value. Output from IsoformSwitchAnalyzeR [(Vitting-Seerup and Sandelin 2019)](https://paperpile.com/c/3gOEAj/swFVh). Vitting-Seerup *et al* define the gene dIF values as the total change within the gene calculated as sum(abs(dIF)) of the transcripts.

| **Ensembl geneID** | **gene name** | **condition 1** | **condition 2** | **gene switch**  **q value** | **gene dIF** |
| --- | --- | --- | --- | --- | --- |
| ENSG00000086848 | ALG9 | Differentiated | Undifferentiated | 1.51E-26 | 0.82194 |
| ENSG00000118363 | SPCS2 | Differentiated | Undifferentiated | 1.83E-21 | 0.38822 |
| ENSG00000221838 | AP4M1 | Differentiated | Undifferentiated | 9.67E-18 | 0.6466 |
| ENSG00000088986 | DYNLL1 | Differentiated | Undifferentiated | 4.99E-12 | 0.46228 |
| ENSG00000167113 | COQ4 | Differentiated | Undifferentiated | 1.25E-11 | 0.39038 |
| ENSG00000112769 | LAMA4 | Differentiated | Undifferentiated | 1.41E-11 | 1.28204 |
| ENSG00000164117 | FBXO8 | Differentiated | Undifferentiated | 1.95E-10 | 0.9781 |
| ENSG00000101460 | MAP1LC3A | Differentiated | Undifferentiated | 1.57E-08 | 0.46222 |
| ENSG00000108384 | RAD51C | Differentiated | Undifferentiated | 4.19E-08 | 0.28446 |
| ENSG00000172053 | QARS | Differentiated | Undifferentiated | 1.08E-07 | 0.61992 |
| ENSG00000169738 | DCXR | Differentiated | Undifferentiated | 2.91E-07 | 0.32198 |
| ENSG00000160789 | LMNA | Differentiated | Undifferentiated | 3.67E-07 | 0.42082 |
| ENSG00000140416 | TPM1 | Differentiated | Undifferentiated | 7.53E-07 | 0.57062 |
| ENSG00000172081 | MOB3A | Differentiated | Undifferentiated | 1.23E-06 | 0.6413 |
| ENSG00000151148 | UBE3B | Differentiated | Undifferentiated | 1.52E-06 | 0.89538 |
| ENSG00000146282 | RARS2 | Differentiated | Undifferentiated | 2.19E-06 | 0.22272 |
| ENSG00000221926 | TRIM16 | Differentiated | Undifferentiated | 6.06E-06 | 0.90108 |
| ENSG00000163738 | MTHFD2L | Differentiated | Undifferentiated | 2.28E-05 | 0.6238 |
| ENSG00000108591 | DRG2 | Differentiated | Undifferentiated | 3.53E-05 | 0.3962 |
| ENSG00000179632 | MAF1 | Differentiated | Undifferentiated | 3.79E-05 | 0.5825 |
| ENSG00000158604 | TMED4 | Differentiated | Undifferentiated | 4.45E-05 | 0.31664 |
| ENSG00000068024 | HDAC4 | Differentiated | Undifferentiated | 9.36E-05 | 1.19254 |
| ENSG00000285437 | POLR2J3 | Differentiated | Undifferentiated | 0.000105381 | 0.46348 |
| ENSG00000125755 | SYMPK | Differentiated | Undifferentiated | 0.000216866 | 0.5643 |
| ENSG00000056972 | TRAF3IP2 | Differentiated | Undifferentiated | 0.000341384 | 0.66476 |
| ENSG00000089486 | CDIP1 | Differentiated | Undifferentiated | 0.000365387 | 0.36804 |
| ENSG00000163053 | SLC16A14 | Differentiated | Undifferentiated | 0.000516673 | 0.85968 |
| ENSG00000171469 | ZNF561 | Differentiated | Undifferentiated | 0.000538662 | 0.55112 |
| ENSG00000055044 | NOP58 | Differentiated | Undifferentiated | 0.000654218 | 0.37566 |
| ENSG00000107317 | PTGDS | Differentiated | Undifferentiated | 0.000728674 | 0.59916 |
| ENSG00000165792 | METTL17 | Differentiated | Undifferentiated | 0.000728674 | 0.57586 |
| ENSG00000127125 | PPCS | Differentiated | Undifferentiated | 0.00074317 | 0.35964 |
| ENSG00000184347 | SLIT3 | Differentiated | Undifferentiated | 0.000875632 | 0.55768 |
| ENSG00000104870 | FCGRT | Differentiated | Undifferentiated | 0.000913016 | 0.51072 |
| ENSG00000180596 | HIST1H2BC | Differentiated | Undifferentiated | 0.000978953 | 0.959 |
| ENSG00000131711 | MAP1B | Differentiated | Undifferentiated | 0.0010522 | 0.47622 |
| ENSG00000179029 | TMEM107 | Differentiated | Undifferentiated | 0.0010522 | 0.28282 |
| ENSG00000140995 | DEF8 | Differentiated | Undifferentiated | 0.001148515 | 0.21586 |
| ENSG00000135931 | ARMC9 | Differentiated | Undifferentiated | 0.001238557 | 0.67826 |
| ENSG00000161800 | RACGAP1 | Differentiated | Undifferentiated | 0.001494161 | 0.46706 |
| ENSG00000146540 | C7orf50 | Differentiated | Undifferentiated | 0.001536413 | 0.35414 |
| ENSG00000183092 | BEGAIN | Differentiated | Undifferentiated | 0.001547641 | 0.68542 |
| ENSG00000123178 | SPRYD7 | Differentiated | Undifferentiated | 0.001966476 | 0.92586 |
| ENSG00000003756 | RBM5 | Differentiated | Undifferentiated | 0.00235666 | 0.55744 |
| ENSG00000166444 | ST5 | Differentiated | Undifferentiated | 0.00235666 | 1.23492 |
| ENSG00000124222 | STX16 | Differentiated | Undifferentiated | 0.002830477 | 0.8726 |
| ENSG00000196704 | AMZ2 | Differentiated | Undifferentiated | 0.002854375 | 0.30688 |
| ENSG00000164466 | SFXN1 | Differentiated | Undifferentiated | 0.00306238 | 0.54652 |
| ENSG00000198408 | OGA | Differentiated | Undifferentiated | 0.003637501 | 0.6726 |
| ENSG00000197372 | ZNF675 | Differentiated | Undifferentiated | 0.004074968 | 0.88006 |
| ENSG00000099330 | OCEL1 | Differentiated | Undifferentiated | 0.004090568 | 0.63212 |
| ENSG00000257591 | ZNF625 | Differentiated | Undifferentiated | 0.00422435 | 0.86368 |
| ENSG00000064490 | RFXANK | Differentiated | Undifferentiated | 0.004622242 | 0.56388 |
| ENSG00000136490 | LIMD2 | Differentiated | Undifferentiated | 0.006030149 | 0.24074 |
| ENSG00000131477 | RAMP2 | Differentiated | Undifferentiated | 0.006540547 | 0.26118 |
| ENSG00000184787 | UBE2G2 | Differentiated | Undifferentiated | 0.006598612 | 0.36004 |
| ENSG00000196465 | MYL6B | Differentiated | Undifferentiated | 0.007142517 | 0.28548 |
| ENSG00000132692 | BCAN | Differentiated | Undifferentiated | 0.007231216 | 0.67606 |
| ENSG00000138606 | SHF | Differentiated | Undifferentiated | 0.007762126 | 0.47386 |
| ENSG00000151353 | TMEM18 | Differentiated | Undifferentiated | 0.007989728 | 0.27162 |
| ENSG00000197122 | SRC | Differentiated | Undifferentiated | 0.008177219 | 0.97768 |
| ENSG00000160013 | PTGIR | Differentiated | Undifferentiated | 0.008905808 | 0.69932 |
| ENSG00000111581 | NUP107 | Differentiated | Undifferentiated | 0.009183614 | 0.4411 |
| ENSG00000197771 | MCMBP | Differentiated | Undifferentiated | 0.009700325 | 0.8666 |
| ENSG00000137434 | C6orf52 | Differentiated | Undifferentiated | 0.009711457 | 0.33414 |
| ENSG00000198551 | ZNF627 | Differentiated | Undifferentiated | 0.00989504 | 0.34544 |
| ENSG00000066379 | ZNRD1 | Differentiated | Undifferentiated | 0.01087531 | 0.3339 |
| ENSG00000062194 | GPBP1 | Differentiated | Undifferentiated | 0.011249117 | 0.53476 |
| ENSG00000166471 | TMEM41B | Differentiated | Undifferentiated | 0.011305356 | 0.63752 |
| ENSG00000174628 | IQCK | Differentiated | Undifferentiated | 0.012469732 | 1.2312 |
| ENSG00000227051 | C14orf132 | Differentiated | Undifferentiated | 0.013453422 | 0.4272 |
| ENSG00000141298 | SSH2 | Differentiated | Undifferentiated | 0.013885209 | 0.22888 |
| ENSG00000121413 | ZSCAN18 | Differentiated | Undifferentiated | 0.014960611 | 0.56362 |
| ENSG00000151617 | EDNRA | Differentiated | Undifferentiated | 0.016912634 | 0.57706 |
| ENSG00000166407 | LMO1 | Differentiated | Undifferentiated | 0.017290737 | 0.44218 |
| ENSG00000100888 | CHD8 | Differentiated | Undifferentiated | 0.01754742 | 0.56924 |
| ENSG00000276234 | TADA2A | Differentiated | Undifferentiated | 0.018780264 | 0.55928 |
| ENSG00000198663 | C6orf89 | Differentiated | Undifferentiated | 0.020507021 | 0.37442 |
| ENSG00000205339 | IPO7 | Differentiated | Undifferentiated | 0.020514719 | 0.54464 |
| ENSG00000136146 | MED4 | Differentiated | Undifferentiated | 0.02127913 | 0.28136 |
| ENSG00000004897 | CDC27 | Differentiated | Undifferentiated | 0.022166596 | 0.68766 |
| ENSG00000119559 | C19orf25 | Differentiated | Undifferentiated | 0.024137326 | 0.31808 |
| ENSG00000060982 | BCAT1 | Differentiated | Undifferentiated | 0.024277036 | 0.38514 |
| ENSG00000196482 | ESRRG | Differentiated | Undifferentiated | 0.027583627 | 0.6638 |
| ENSG00000139182 | CLSTN3 | Differentiated | Undifferentiated | 0.027739353 | 0.49136 |
| ENSG00000204653 | ASPDH | Differentiated | Undifferentiated | 0.028996139 | 0.6774 |
| ENSG00000106635 | BCL7B | Differentiated | Undifferentiated | 0.029720469 | 0.54668 |
| ENSG00000144401 | METTL21A | Differentiated | Undifferentiated | 0.029949427 | 0.33934 |
| ENSG00000135090 | TAOK3 | Differentiated | Undifferentiated | 0.033871798 | 0.71412 |
| ENSG00000149292 | TTC12 | Differentiated | Undifferentiated | 0.033913158 | 0.66326 |
| ENSG00000171953 | ATPAF2 | Differentiated | Undifferentiated | 0.034218455 | 0.377 |
| ENSG00000080986 | NDC80 | Differentiated | Undifferentiated | 0.034297085 | 0.46102 |
| ENSG00000105321 | CCDC9 | Differentiated | Undifferentiated | 0.034903122 | 0.92352 |
| ENSG00000135541 | AHI1 | Differentiated | Undifferentiated | 0.035365825 | 0.85788 |
| ENSG00000122490 | PQLC1 | Differentiated | Undifferentiated | 0.036240151 | 0.47884 |
| ENSG00000174353 | STAG3L3 | Differentiated | Undifferentiated | 0.038682053 | 0.3354 |
| ENSG00000140939 | NOL3 | Differentiated | Undifferentiated | 0.04412664 | 0.33764 |
| ENSG00000147548 | NSD3 | Differentiated | Undifferentiated | 0.044278588 | 0.48134 |
| ENSG00000104231 | ZFAND1 | Differentiated | Undifferentiated | 0.044308667 | 0.33178 |
| ENSG00000107951 | MTPAP | Differentiated | Undifferentiated | 0.044598461 | 0.24312 |
| ENSG00000166398 | KIAA0355 | Differentiated | Undifferentiated | 0.046626246 | 0.47908 |
| ENSG00000259431 | THTPA | Differentiated | Undifferentiated | 0.047528021 | 0.62722 |
| ENSG00000135249 | RINT1 | Differentiated | Undifferentiated | 0.047949428 | 0.81788 |
| ENSG00000136574 | GATA4 | Differentiated | Undifferentiated | 0.048524879 | 0.3974 |

**Table S5** *CACNA2D2* RT-PCR validation primers designed with Primer-BLAST [(Ye et al. 2012)](https://paperpile.com/c/3gOEAj/MncP0). All primer sets used the same reverse primer. SC = self-complementarity and S3’C = Self 3’ complementarity.

| **Primers**  **(product size)** | **Sequence (5'->3')** | **Length** | **Start** | **Stop** | **Tm** | **GC%** | **SC** | **S3'C** |
| --- | --- | --- | --- | --- | --- | --- | --- | --- |
| Novel-F (272) | AAGCCTGGAGTGCATGCTGAAT | 22 | 8 | 29 | 62.87 | 50 | 7 | 3 |
| Junc-F (261) | GCATGCTGAATGGCCACTGT | 20 | 19 | 38 | 61.6 | 55 | 6 | 3 |
| Ctrl-F (214) | GGCCCTGGAGAATGTGTTCA | 20 | 66 | 85 | 59.96 | 55 | 4 | 2 |
| Reverse primer | GCCACTCACATCCACGATGA | 20 | 279 | 260 | 60.11 | 55 | 5 | 3 |

**Table S6** List of N=333 novel transcripts possessing putatively novel transcription start sites and which display CAGEseq peak overlap (± 500bp). Chromosome, overlap interval start and end and novel TALON transcript ID are provided.

| Chromosome | Interval start | Interval end | Transcript ID |
| --- | --- | --- | --- |
| chr1 | 1718491 | 1718991 | TALONT000216863 |
| chr1 | 1720499 | 1720999 | TALONT000216861 |
| chr1 | 8926419 | 8926919 | TALONT000252295 |
| chr1 | 32687533 | 32688033 | TALONT000534035 |
| chr1 | 44446318 | 44446818 | TALONT000667860 |
| chr1 | 54411524 | 54412024 | TALONT000762103 |
| chr1 | 109506071 | 109506571 | TALONT000956540 |
| chr1 | 109506071 | 109506571 | TALONT000956567 |
| chr1 | 151226707 | 151227207 | TALONT001253434 |
| chr1 | 153641012 | 153641512 | TALONT001282209 |
| chr1 | 156308032 | 156308532 | TALONT001495992 |
| chr1 | 156713996 | 156714496 | TALONT001532571 |
| chr1 | 163131757 | 163132257 | TALONT001557153 |
| chr1 | 163131765 | 163132265 | TALONT001557167 |
| chr1 | 163172703 | 163173203 | TALONT001557179 |
| chr1 | 163172703 | 163173203 | TALONT001557187 |
| chr1 | 163172703 | 163173203 | TALONT001557824 |
| chr1 | 163172703 | 163173203 | TALONT001558055 |
| chr1 | 163172703 | 163173203 | TALONT001558338 |
| chr1 | 163172703 | 163173203 | TALONT001558418 |
| chr1 | 169763670 | 169764170 | TALONT001565538 |
| chr1 | 212588186 | 212588686 | TALONT001585346 |
| chr1 | 214776018 | 214776518 | TALONT001586884 |
| chr1 | 214776018 | 214776518 | TALONT001586885 |
| chr1 | 225590791 | 225591291 | TALONT001591382 |
| chr1 | 228284791 | 228285291 | TALONT001595673 |
| chr1 | 235812989 | 235813489 | TALONT001600194 |
| chr1 | 155225269 | 155225769 | TALONT000363433 |
| chr1 | 155225269 | 155225769 | TALONT000363440 |
| chr10 | 1033892 | 1034392 | TALONT001606287 |
| chr10 | 17270649 | 17271149 | TALONT001613282 |
| chr10 | 17270649 | 17271149 | TALONT001613285 |
| chr10 | 17270649 | 17271149 | TALONT001613287 |
| chr10 | 17270649 | 17271149 | TALONT001613289 |
| chr10 | 17270649 | 17271149 | TALONT001613291 |
| chr10 | 17270649 | 17271149 | TALONT001613293 |
| chr10 | 17270755 | 17271255 | TALONT001613298 |
| chr10 | 17270755 | 17271255 | TALONT001613314 |
| chr10 | 17270770 | 17271270 | TALONT001613406 |
| chr10 | 33200454 | 33200954 | TALONT001619327 |
| chr10 | 73579539 | 73580039 | TALONT001632282 |
| chr10 | 73581763 | 73582263 | TALONT001632237 |
| chr10 | 73610976 | 73611476 | TALONT001632314 |
| chr10 | 75160614 | 75161114 | TALONT001635861 |
| chr10 | 85898766 | 85899266 | TALONT001639932 |
| chr10 | 85898766 | 85899266 | TALONT001639938 |
| chr10 | 90751112 | 90751612 | TALONT001641623 |
| chr10 | 120801758 | 120802258 | TALONT001657624 |
| chr10 | 120801758 | 120802258 | TALONT001657628 |
| chr10 | 120810185 | 120810685 | TALONT001657621 |
| chr10 | 129900862 | 129901362 | TALONT001662124 |
| chr11 | 819073 | 819573 | TALONT001665115 |
| chr11 | 819073 | 819573 | TALONT001665116 |
| chr11 | 10830466 | 10830966 | TALONT001674707 |
| chr11 | 10830466 | 10830966 | TALONT001674917 |
| chr11 | 57102713 | 57103213 | TALONT001691299 |
| chr11 | 61632609 | 61633109 | TALONT001696298 |
| chr11 | 64948195 | 64948695 | TALONT001703613 |
| chr11 | 65768960 | 65769460 | TALONT001706199 |
| chr11 | 73881648 | 73882148 | TALONT001715600 |
| chr11 | 118971536 | 118972036 | TALONT001732216 |
| chr12 | 6643182 | 6643682 | TALONT000284409 |
| chr12 | 6645159 | 6645659 | TALONT000299709 |
| chr12 | 6645159 | 6645659 | TALONT000299738 |
| chr12 | 6645349 | 6645849 | TALONT000299883 |
| chr12 | 16034799 | 16035299 | TALONT000356165 |
| chr12 | 16034812 | 16035312 | TALONT000356180 |
| chr12 | 16034812 | 16035312 | TALONT000356192 |
| chr12 | 16034812 | 16035312 | TALONT000356199 |
| chr12 | 16034812 | 16035312 | TALONT000356203 |
| chr12 | 16034812 | 16035312 | TALONT000356223 |
| chr12 | 16034821 | 16035321 | TALONT000356554 |
| chr12 | 49523097 | 49523597 | TALONT000420786 |
| chr12 | 49523166 | 49523666 | TALONT000422318 |
| chr12 | 49580138 | 49580638 | TALONT000545335 |
| chr12 | 49580241 | 49580741 | TALONT000556982 |
| chr12 | 54674003 | 54674503 | TALONT000764864 |
| chr12 | 54674003 | 54674503 | TALONT000765131 |
| chr12 | 56497897 | 56498397 | TALONT000826866 |
| chr12 | 56497897 | 56498397 | TALONT000826874 |
| chr12 | 57066793 | 57067293 | TALONT000905310 |
| chr12 | 89743337 | 89743837 | TALONT001027321 |
| chr12 | 118573330 | 118573830 | TALONT001203100 |
| chr12 | 125399156 | 125399656 | TALONT001295910 |
| chr12 | 125399193 | 125399693 | TALONT001307517 |
| chr12 | 31479044 | 31479544 | TALONT000213640 |
| chr12 | 31479044 | 31479544 | TALONT000213649 |
| chr13 | 48669195 | 48669695 | TALONT000286157 |
| chr14 | 35182602 | 35183102 | TALONT000274851 |
| chr14 | 89306706 | 89307206 | TALONT000477826 |
| chr14 | 89306771 | 89307271 | TALONT000478166 |
| chr14 | 90870532 | 90871032 | TALONT000492998 |
| chr14 | 102549633 | 102550133 | TALONT000604731 |
| chr14 | 102550029 | 102550529 | TALONT000604814 |
| chr14 | 102551767 | 102552267 | TALONT000604788 |
| chr14 | 93388964 | 93389464 | TALONT000221836 |
| chr14 | 93651225 | 93651725 | TALONT000222393 |
| chr15 | 44038081 | 44038581 | TALONT000286024 |
| chr15 | 44038081 | 44038581 | TALONT000286029 |
| chr15 | 51973153 | 51973653 | TALONT000326204 |
| chr15 | 45003210 | 45003710 | TALONT000213580 |
| chr16 | 28857592 | 28858092 | TALONT000368878 |
| chr16 | 28857669 | 28858169 | TALONT000368904 |
| chr16 | 28857669 | 28858169 | TALONT000368973 |
| chr16 | 49669836 | 49670336 | TALONT000469961 |
| chr16 | 55516320 | 55516820 | TALONT000478674 |
| chr16 | 55525212 | 55525712 | TALONT000479028 |
| chr16 | 56553965 | 56554465 | TALONT000484349 |
| chr16 | 57219910 | 57220410 | TALONT000489972 |
| chr16 | 57219910 | 57220410 | TALONT000489975 |
| chr17 | 685459 | 685959 | TALONT000772865 |
| chr17 | 1972087 | 1972587 | TALONT000783578 |
| chr17 | 4853590 | 4854090 | TALONT000795842 |
| chr17 | 7475581 | 7476081 | TALONT000817207 |
| chr17 | 7475641 | 7476141 | TALONT000817241 |
| chr17 | 17942491 | 17942991 | TALONT000858932 |
| chr17 | 19288514 | 19289014 | TALONT000868022 |
| chr17 | 19289776 | 19290276 | TALONT000867277 |
| chr17 | 30813993 | 30814493 | TALONT000921133 |
| chr17 | 33913786 | 33914286 | TALONT000925624 |
| chr17 | 33913786 | 33914286 | TALONT000925630 |
| chr17 | 33913786 | 33914286 | TALONT000925634 |
| chr17 | 33913786 | 33914286 | TALONT000925661 |
| chr17 | 40169643 | 40170143 | TALONT000987370 |
| chr17 | 61903974 | 61904474 | TALONT001225521 |
| chr17 | 73000108 | 73000608 | TALONT001339402 |
| chr17 | 74264329 | 74264829 | TALONT001409254 |
| chr17 | 74556869 | 74557369 | TALONT001433561 |
| chr17 | 74556869 | 74557369 | TALONT001433563 |
| chr17 | 79478029 | 79478529 | TALONT001524888 |
| chr17 | 79478110 | 79478610 | TALONT001499854 |
| chr17 | 79479293 | 79479793 | TALONT001500555 |
| chr17 | 79525814 | 79526314 | TALONT001550177 |
| chr17 | 79894736 | 79895236 | TALONT001554087 |
| chr17 | 34890795 | 34891316 | TALONT000231262 |
| chr17 | 35305736 | 35306236 | TALONT000231713 |
| chr17 | 35849464 | 35849964 | TALONT000232180 |
| chr17 | 35971276 | 35971776 | TALONT000232545 |
| chr19 | 2762641 | 2763141 | TALONT000307130 |
| chr19 | 3979326 | 3979826 | TALONT000313530 |
| chr19 | 4861422 | 4861922 | TALONT000345447 |
| chr19 | 12254511 | 12255011 | TALONT000426042 |
| chr19 | 15235879 | 15236379 | TALONT000498042 |
| chr19 | 15236194 | 15236694 | TALONT000498049 |
| chr19 | 16186746 | 16187246 | TALONT000502544 |
| chr19 | 36358949 | 36359449 | TALONT000622962 |
| chr19 | 37829966 | 37830466 | TALONT000644688 |
| chr19 | 41808752 | 41809252 | TALONT000696792 |
| chr19 | 41808752 | 41809252 | TALONT000696793 |
| chr19 | 47285692 | 47286192 | TALONT000779972 |
| chr19 | 47285692 | 47286192 | TALONT000780407 |
| chr19 | 49403542 | 49404042 | TALONT000833979 |
| chr19 | 49863337 | 49863837 | TALONT000847477 |
| chr19 | 50432738 | 50433238 | TALONT000878134 |
| chr19 | 54376290 | 54376790 | TALONT000923114 |
| chr2 | 9563261 | 9563761 | TALONT000413546 |
| chr2 | 9563261 | 9563761 | TALONT000413575 |
| chr2 | 9563261 | 9563761 | TALONT000413592 |
| chr2 | 10584392 | 10584892 | TALONT000430285 |
| chr2 | 10942765 | 10943265 | TALONT000433115 |
| chr2 | 10942765 | 10943265 | TALONT000433970 |
| chr2 | 11622427 | 11622927 | TALONT000443015 |
| chr2 | 15734768 | 15735268 | TALONT000448324 |
| chr2 | 20251372 | 20251872 | TALONT000450988 |
| chr2 | 20402695 | 20403195 | TALONT000453803 |
| chr2 | 113402939 | 113403439 | TALONT000837074 |
| chr2 | 114341004 | 114341504 | TALONT000842406 |
| chr2 | 153573944 | 153574444 | TALONT000927595 |
| chr2 | 189873212 | 189873712 | TALONT001079211 |
| chr2 | 217276956 | 217277456 | TALONT001264164 |
| chr2 | 220405363 | 220405863 | TALONT001321926 |
| chr2 | 232572733 | 232573233 | TALONT001378949 |
| chr20 | 10288065 | 10288565 | TALONT001446440 |
| chr20 | 35807021 | 35807521 | TALONT001476710 |
| chr20 | 36147751 | 36148251 | TALONT001479309 |
| chr20 | 47804875 | 47805375 | TALONT001496141 |
| chr21 | 46276278 | 46276778 | TALONT000458263 |
| chr22 | 24819098 | 24819598 | TALONT000422415 |
| chr22 | 24819098 | 24819598 | TALONT000422416 |
| chr22 | 24819098 | 24819598 | TALONT000422427 |
| chr22 | 24828598 | 24829098 | TALONT000422617 |
| chr22 | 24828598 | 24829098 | TALONT000422619 |
| chr22 | 46067208 | 46067708 | TALONT000531803 |
| chr22 | 46067210 | 46067710 | TALONT000531971 |
| chr3 | 37316635 | 37317135 | TALONT000593850 |
| chr3 | 49055985 | 49056485 | TALONT000648957 |
| chr3 | 49094475 | 49094975 | TALONT000655690 |
| chr3 | 49094855 | 49095355 | TALONT000655714 |
| chr3 | 52001449 | 52001949 | TALONT000712136 |
| chr3 | 98235472 | 98235972 | TALONT000828694 |
| chr3 | 112358762 | 112359262 | TALONT000881051 |
| chr4 | 77987547 | 77988047 | TALONT000674931 |
| chr4 | 103748000 | 103748500 | TALONT000715384 |
| chr4 | 103748891 | 103749391 | TALONT000715438 |
| chr4 | 154257013 | 154257513 | TALONT000818898 |
| chr4 | 52715 | 53215 | TALONT001279809 |
| chr5 | 10257722 | 10258222 | TALONT000680319 |
| chr5 | 10257722 | 10258222 | TALONT000680320 |
| chr5 | 71489303 | 71489803 | TALONT000755050 |
| chr5 | 73931099 | 73931599 | TALONT000765520 |
| chr5 | 77655830 | 77656330 | TALONT000774140 |
| chr5 | 154193990 | 154194490 | TALONT001068956 |
| chr5 | 179233451 | 179233951 | TALONT001194907 |
| chr5 | 179233451 | 179233951 | TALONT001194908 |
| chr5 | 180258104 | 180258604 | TALONT001208200 |
| chr6 | 3226502 | 3227002 | TALONT000700197 |
| chr6 | 3227881 | 3228381 | TALONT000700135 |
| chr6 | 24719067 | 24719567 | TALONT000729969 |
| chr6 | 36653027 | 36653527 | TALONT000785873 |
| chr6 | 42984367 | 42984867 | TALONT000808148 |
| chr6 | 44213854 | 44214354 | TALONT000820111 |
| chr6 | 44214280 | 44214780 | TALONT000820130 |
| chr6 | 44214280 | 44214780 | TALONT000820151 |
| chr6 | 44214313 | 44214813 | TALONT000820160 |
| chr6 | 44214313 | 44214813 | TALONT000820171 |
| chr6 | 44214332 | 44214832 | TALONT000820255 |
| chr6 | 44214332 | 44214832 | TALONT000820274 |
| chr6 | 44214332 | 44214832 | TALONT000820310 |
| chr6 | 44214332 | 44214832 | TALONT000820464 |
| chr6 | 44214332 | 44214832 | TALONT000820587 |
| chr6 | 44214332 | 44214832 | TALONT000821701 |
| chr6 | 44214332 | 44214832 | TALONT000821795 |
| chr6 | 44214332 | 44214832 | TALONT000821809 |
| chr6 | 44214332 | 44214832 | TALONT000822155 |
| chr6 | 44214332 | 44214832 | TALONT000822457 |
| chr6 | 44215853 | 44216353 | TALONT000841952 |
| chr6 | 44215853 | 44216353 | TALONT000841953 |
| chr6 | 44216042 | 44216542 | TALONT000842418 |
| chr6 | 44217257 | 44217757 | TALONT000842923 |
| chr6 | 44217257 | 44217757 | TALONT000842935 |
| chr6 | 44217527 | 44218027 | TALONT000843250 |
| chr6 | 44217527 | 44218027 | TALONT000843259 |
| chr6 | 44218654 | 44219154 | TALONT000844467 |
| chr6 | 44219233 | 44219733 | TALONT000844903 |
| chr6 | 64281416 | 64281916 | TALONT000868363 |
| chr6 | 74228245 | 74228745 | TALONT000884918 |
| chr6 | 74228285 | 74228785 | TALONT000884836 |
| chr6 | 74228516 | 74229016 | TALONT000884896 |
| chr6 | 74228516 | 74229016 | TALONT000884923 |
| chr6 | 114181104 | 114181604 | TALONT001025753 |
| chr6 | 151669820 | 151670320 | TALONT001096618 |
| chr6 | 159204609 | 159205109 | TALONT001116591 |
| chr6 | 31795213 | 31795713 | TALONT001160973 |
| chr6 | 31509779 | 31510279 | TALONT001175021 |
| chr6 | 33290684 | 33291184 | TALONT001193017 |
| chr6 | 33421819 | 33422319 | TALONT001208400 |
| chr7 | 5568301 | 5568801 | TALONT000900939 |
| chr7 | 5568337 | 5568837 | TALONT000900560 |
| chr7 | 5569006 | 5569506 | TALONT000900477 |
| chr7 | 5569030 | 5569530 | TALONT000900442 |
| chr7 | 5570229 | 5570729 | TALONT000900479 |
| chr7 | 19156794 | 19157294 | TALONT000950247 |
| chr7 | 26236494 | 26236994 | TALONT000969433 |
| chr7 | 26237015 | 26237515 | TALONT000969507 |
| chr7 | 26240354 | 26240854 | TALONT000969361 |
| chr7 | 26240354 | 26240854 | TALONT000969378 |
| chr7 | 26240363 | 26240863 | TALONT000969426 |
| chr7 | 26240363 | 26240863 | TALONT000969464 |
| chr7 | 26240363 | 26240863 | TALONT000969831 |
| chr7 | 26240850 | 26241350 | TALONT000977823 |
| chr7 | 35840298 | 35840798 | TALONT000994931 |
| chr7 | 47316960 | 47317460 | TALONT001049250 |
| chr7 | 56101846 | 56102346 | TALONT001063536 |
| chr7 | 72936292 | 72936792 | TALONT001114767 |
| chr7 | 97501719 | 97502219 | TALONT001217699 |
| chr7 | 97501747 | 97502247 | TALONT001217731 |
| chr7 | 97501760 | 97502260 | TALONT001217704 |
| chr7 | 97501778 | 97502278 | TALONT001217706 |
| chr7 | 97501778 | 97502278 | TALONT001217771 |
| chr7 | 97501778 | 97502278 | TALONT001217860 |
| chr7 | 97501782 | 97502282 | TALONT001217702 |
| chr7 | 97501782 | 97502282 | TALONT001220883 |
| chr7 | 99646894 | 99647394 | TALONT001260578 |
| chr7 | 99696825 | 99697325 | TALONT001266073 |
| chr7 | 99696825 | 99697325 | TALONT001266334 |
| chr7 | 99699009 | 99699509 | TALONT001266282 |
| chr7 | 99699014 | 99699514 | TALONT001266278 |
| chr7 | 99933701 | 99934201 | TALONT001286465 |
| chr7 | 99933701 | 99934201 | TALONT001286485 |
| chr7 | 99933701 | 99934201 | TALONT001286517 |
| chr7 | 100199474 | 100199974 | TALONT001289202 |
| chr7 | 102312087 | 102312587 | TALONT001332319 |
| chr7 | 132720679 | 132721179 | TALONT001437977 |
| chr7 | 143079392 | 143079892 | TALONT001512943 |
| chr7 | 143079393 | 143079893 | TALONT001512949 |
| chr7 | 148395354 | 148395854 | TALONT001515575 |
| chr8 | 17434144 | 17434644 | TALONT001025887 |
| chr8 | 56685493 | 56685993 | TALONT001079290 |
| chr8 | 75896235 | 75896735 | TALONT001104271 |
| chr8 | 117887039 | 117887539 | TALONT001188087 |
| chr8 | 128749408 | 128749908 | TALONT001203558 |
| chr9 | 32550965 | 32551465 | TALONT001242609 |
| chr9 | 100745105 | 100745605 | TALONT001299287 |
| chr9 | 128001459 | 128001959 | TALONT001347524 |
| chr9 | 128001484 | 128001984 | TALONT001347509 |
| chr9 | 128002968 | 128003468 | TALONT001347548 |
| chr9 | 136500967 | 136501467 | TALONT001468074 |
| chr9 | 136500967 | 136501467 | TALONT001468097 |
| chr9 | 136500967 | 136501467 | TALONT001468106 |
| chr9 | 136500967 | 136501467 | TALONT001468363 |
| chr9 | 136500967 | 136501467 | TALONT001468372 |
| chr9 | 136500967 | 136501467 | TALONT001468393 |
| chr9 | 136500967 | 136501467 | TALONT001468432 |
| chr9 | 136500967 | 136501467 | TALONT001468442 |
| chr9 | 136500967 | 136501467 | TALONT001468449 |
| chr9 | 136500967 | 136501467 | TALONT001468473 |
| chr9 | 136500967 | 136501467 | TALONT001468478 |
| chr9 | 136500967 | 136501467 | TALONT001468492 |
| chr9 | 136500967 | 136501467 | TALONT001468742 |
| chr9 | 136500967 | 136501467 | TALONT001468766 |
| chr9 | 136500967 | 136501467 | TALONT001468853 |
| chr9 | 136500967 | 136501467 | TALONT001469014 |
| chr9 | 136500978 | 136501478 | TALONT001469134 |
| chr9 | 139377496 | 139377996 | TALONT001496298 |
| chrX | 1508495 | 1508995 | TALONT001297471 |
| chrX | 21958293 | 21958793 | TALONT001311103 |
| chrX | 23855437 | 23855937 | TALONT001313297 |
| chrX | 51636221 | 51636721 | TALONT001334152 |
| chrX | 51636838 | 51637338 | TALONT001336538 |
| chrX | 51636838 | 51637338 | TALONT001336539 |
| chrX | 54833621 | 54834121 | TALONT001343253 |
| chrX | 54833621 | 54834121 | TALONT001343296 |
| chrX | 62571222 | 62571722 | TALONT001347777 |
| chrX | 70502964 | 70503464 | TALONT001355954 |
| chrX | 70502964 | 70503464 | TALONT001355966 |
| chrX | 71492062 | 71492562 | TALONT001365609 |
| chrX | 100662695 | 100663195 | TALONT001389016 |
| chrX | 100662695 | 100663195 | TALONT001389018 |
| chrX | 118763347 | 118763847 | TALONT001404875 |
| chrX | 130215142 | 130215642 | TALONT001421153 |
| chrX | 130215142 | 130215642 | TALONT001421205 |
| chrX | 130217537 | 130218037 | TALONT001426522 |
| chrX | 131351693 | 131352193 | TALONT001427813 |

**Table S7** Overview of genome-wide alternative splicing events between cell states during differentiation of SHSY5Y cells. IF = Isoform Fraction. 1 = differentiated, 2 = undifferentiated. Each row is a comparison between differentiated vs undifferentiated cells. A5 & A3 = alternative donor and acceptor sites, IR = intron retention, ATSS = alternative first exon, ATTS = alternative last exon, MEE = mutually exclusive exon, ES = exon skipping, MES = multiple exon skipping. Produced with IsoformSwitchAnalyzeR.

| **event** | **isoform**  **feature** | **isoforms n** | **events**  **n** | **median IF1** | **median IF2** | **median DIF** | **wilcox**  **Pval** | **wilcox**  **Qval** | **significance** |
| --- | --- | --- | --- | --- | --- | --- | --- | --- | --- |
| A3 | With | 8024 | 10421 | 0.0889 | 0.09218 | 0.00328 | 4.36E-01 | 5.36E-01 | ns |
| A3 | Without | 20839 | - | 0.07984 | 0.084 | 0.00416 | 2.61E-04 | 8.34E-04 | *** |
| A5 | With | 10059 | 13874 | 0.08866 | 0.09014 | 0.00148 | 4.95E-01 | 5.65E-01 | ns |
| A5 | Without | 18804 | - | 0.07891 | 0.08414 | 0.00523 | 1.27E-04 | 6.79E-04 | *** |
| ATSS | With | 21203 | 21203 | 0.06146 | 0.0655 | 0.00404 | 7.92E-06 | 6.34E-05 | *** |
| ATSS | Without | 7660 | - | 0.22275 | 0.22702 | 0.00427 | 9.67E-01 | 9.67E-01 | ns |
| ATTS | With | 19992 | 19992 | 0.05798 | 0.06225 | 0.00427 | 1.36E-06 | 2.18E-05 | *** |
| ATTS | Without | 8871 | - | 0.20932 | 0.20766 | -0.00166 | 7.26E-01 | 7.74E-01 | ns |
| ES | With | 11228 | 14996 | 0.08529 | 0.08731 | 0.00202 | 3.77E-01 | 5.32E-01 | ns |
| ES | Without | 17635 | - | 0.08064 | 0.08544 | 0.0048 | 1.88E-04 | 7.54E-04 | *** |
| IR | With | 3074 | 3833 | 0.05618 | 0.06222 | 0.00604 | 1.17E-02 | 2.09E-02 | * |
| IR | Without | 25789 | - | 0.0873 | 0.09076 | 0.00346 | 3.68E-03 | 7.36E-03 | * |
| MEE | With | 330 | 354 | 0.05016 | 0.06189 | 0.01173 | 3.99E-01 | 5.32E-01 | ns |
| MEE | Without | 28533 | - | 0.08308 | 0.08672 | 0.00364 | 6.01E-04 | 1.56E-03 | * |
| MES | With | 5627 | 6346 | 0.06492 | 0.06658 | 0.00166 | 3.66E-01 | 5.32E-01 | ns |
| MES | Without | 23236 | - | 0.08846 | 0.09343 | 0.00497 | 6.84E-04 | 1.56E-03 | * |

**SUPPLEMENTARY CITATIONS**

[Broad Institute. 2019. *Picard Toolkit*.](http://paperpile.com/b/3gOEAj/Z0IF9) <http://broadinstitute.github.io/picard/>[.](http://paperpile.com/b/3gOEAj/Z0IF9)

[Kelley, Lawrence A., Stefans Mezulis, Christopher M. Yates, Mark N. Wass, and Michael J. E. Sternberg. 2015. “The Phyre2 Web Portal for Protein Modeling, Prediction and Analysis.” *Nature Protocols* 10 (6): 845–58.](http://paperpile.com/b/3gOEAj/H86zq)

[Vitting-Seerup, Kristoffer, and Albin Sandelin. 2019. “IsoformSwitchAnalyzeR: Analysis of Changes in Genome-Wide Patterns of Alternative Splicing and Its Functional Consequences.” *Bioinformatics*  35 (21): 4469–71.](http://paperpile.com/b/3gOEAj/swFVh)

[Wong, Ted, Ira W. Deveson, Simon A. Hardwick, and Tim R. Mercer. 2017. “ANAQUIN: A Software Toolkit for the Analysis of Spike-in Controls for next Generation Sequencing.” *Bioinformatics*  33 (11): 1723–24.](http://paperpile.com/b/3gOEAj/e1p37)

[Wyman, Dana, Gabriela Balderrama-Gutierrez, Fairlie Reese, Shan Jiang, Sorena Rahmanian, Stefania Forner, Dina Matheos, et al. 2020. “A Technology-Agnostic Long-Read Analysis Pipeline for Transcriptome Discovery and Quantification.” *bioRxiv*. https://doi.org/](http://paperpile.com/b/3gOEAj/W091i)[10.1101/672931](http://dx.doi.org/10.1101/672931)[.](http://paperpile.com/b/3gOEAj/W091i)

[Ye, Jian, George Coulouris, Irena Zaretskaya, Ioana Cutcutache, Steve Rozen, and Thomas L. Madden. 2012. “Primer-BLAST: A Tool to Design Target-Specific Primers for Polymerase Chain Reaction.” *BMC Bioinformatics* 13 (June): 134.](http://paperpile.com/b/3gOEAj/MncP0)
